# Stereoselective Access to Antimelanoma Agents by Hybridization and Dimerization of Dihydroartemisinin and Artesunic acid

**DOI:** 10.1002/cmdc.202100196

**Published:** 2021-05-07

**Authors:** Lorenzo Botta, Silvia Cesarini, Claudio Zippilli, Silvia Filippi, Bruno Mattia Bizzarri, Maria Camilla Baratto, Rebecca Pogni, Raffaele Saladino

**Affiliations:** ^1^ Department of Biological and Ecological Sciences Univeristy of Viterbo Via S.C. De Lellis s.n.c. 01100 Viterbo Italy; ^2^ Department of Biotechnology Chemistry and Pharmacy University of Siena via Aldo Moro 2 53100 Siena Italy

**Keywords:** artemisinin hybrids and dimers, stereoselectivity, regioselectivity, EPR spectroscopy, antimelanoma activity

## Abstract

A library of five hybrids and six dimers of dihydroartemisinin and artesunic acid has been synthetized in a stereo‐controlled manner and evaluated for the anticancer activity against metastatic melanoma cell line (RPMI7951). Among novel derivatives, three artesunic acid dimers showed antimelanoma activity and cancer selectivity, being not toxic on normal human fibroblast (C3PV) cell line. Among the three dimers, the one bearing 4‐hydroxybenzyl alcohol as a spacer showed no cytotoxic effect (CC_50_>300 μM) and high antimelanoma activity (IC_50_=0.05 μM), which was two orders of magnitude higher than that of parent artesunic acid, and of the same order of commercial drug paclitaxel. In addition, this dimer showed cancer‐type selectivity towards melanoma compared to prostate (PC3) and breast (MDA‐MB‐231) tumors. The occurrence of a radical mechanism was hypothesized by DFO and EPR analyses. Qualitative structure activity relationships highlighted the role of artesunic acid scaffold in the control of toxicity and antimelanoma activity.

## Graphical abstract

**Accessing antimelanoma agents**: The artesunic acid dimer **22‐α**,**α**, bearing 4‐hydroxybenzyl alcohol as linker, showed higher antimelanoma effect and lower cytotoxicity compared to the parent dimer **8** bearing 4‐hydroxyphenetyl alcohol (tyrosol), highlighting the importance of a methylene group in the final effect. In addition, a melanoma cancer‐type selectivity was registered as well as a correlation between the presence of iron and the biological activity.

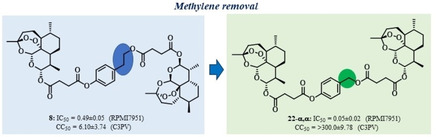

## Introduction

**Figure 1:**
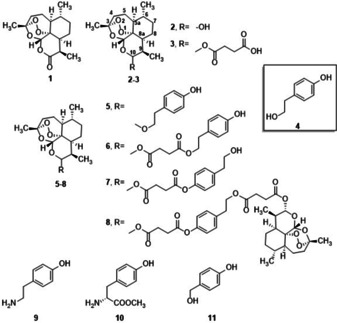
Structures of artemisinin **1**, dihydroartemisinin (DHA) **2**, artesunic acid (ART) **3**, tyrosol **4**, tyramine **9**, L‐tyrosine methyl ester **10** and 4‐hydroxybenzyl alcohol **11** and of representative hybrid and dimer derivatives of DHA and ART **5**–**8**, active against complementary metastatic melanoma cancer cell lines.

Malignant melanoma is a degenerative transformation of melanocytes associated to constant growing incidence, high mortality rate^[1]^ and drug resistance.^[2]^ Conventional anticancer drugs such as cisplatin, dacarbazine, temozolomide, and paclitaxel showed low selectivity against melanoma with concomitant emergence of detrimental side effects.^[3]^ The hybridization and dimerization (HD) approach received an increasing interest in order to overcome drug resistance and produce more active and selective anticancer compounds.^[4]^ Within this procedure, different scaffolds are linked together to give a hybrid derivative, or in alternative, the same bioactive scaffold is repeated twice in a dimer, in order to increase the pharmacological activity and pharmacokinetic profile of the molecule.^[5]^ The HD process proved to be particularly effective when applied to natural products,^[6]^ as in the case of the polycyclic sesquiterpene artemisinin **1** (Figure 1).^[7]^ This compound and its derivatives, dihydroartemisinin (DHA) **2** and artesunic acid (ART) **3** (Figure 1), were used in the synthesis of hybrid and dimer derivatives^[8]^ with antimalarial,^[9]^ antiviral^[10]^ and anticancer activity.^[11]^

Recently, we reported the synthesis of a first library of hybrid and dimer derivatives of **2** and **3** containing phytotherapeutic natural products as additional scaffold and spacer moieties.^[12]^

From this first library, tyrosol (4‐hydroxy phenethylalcohol, **4**) derivatives **5**–**8** (Figure 1) were active against three complementary metastatic melanoma cancer cell lines SK‐MEL3, SK‐MEL24, and RPMI‐7951, respectively. Tyrosol is a C‐2 phenol derivative recovered from the leaf extract of Olea europaea L. and characterized by antioxidant and anticancer effects.^[13]^ In accordance with the biological mechanism reported for **1**–**3**,^[14]^ EPR analysis suggested the formation of C‐centered radical intermediates in the activity of compounds **5**–**8**, even if the inhibitory effect against Human DNA Topoisomerase 1 cannot be completely ruled‐out.^[15]^ The exploration of the chemical space around the DHA and ART scaffolds in **5**–**8** was realized by linking with the primary alcohol moiety (hybrids **5** and **6**), or in alternative, the phenol group (hybrid **7**). In the case of dimer **8**, both hydroxyls were involved (Figure 1). This chemical diversity effectively controlled the antimelanoma activity. For example, the hybrid **7** showed antimelanoma activity against RPMI‐7951 cell line (IC_50_=0.09±0.03) higher than **6** (IC_50_=8.34±3.06), while the lack of the succinate spacer in hybrid **5** (IC_50_=0.33±0.08), or the involvement of both hydroxyl groups in dimer **8** (IC_50_=1.37±0.13), afforded an intermediate behavior.^[12]^

We report here the synthesis of a novel library of DHA and ART derivatives by stereoselective synthesis of DHA/tyrosol hybrids, and the use of tyramine **9**, L‐tyrosine methyl ester **10** and 4‐hydroxybenzyl alcohol **11**, as nitrogen containing and smaller side‐chain analogues of tyrosol (Figure 1). Compounds **9**–**11**, in addition to phthalic acid and 1,4‐butandiol, were also used as spacers for the stereoselective preparation of six novel DHA and ART dimers. The novel products have been tested against RPMI 7951 metastatic melanoma cancer cell line, showing from acceptable to good IC_50_ values. In particular, dimer **22‐α**,**α** showed no cytotoxic activity (CC_50_=>300 μM) on C3PV cell line and high antimelanoma activity (IC_50_=0.05 μM), which was found to be two orders of magnitude higher than that of the parent artesunic acid (IC_50_=1.08 μM), and of the same range of magnitude than commercial drug paclitaxel (IC_50_=0.013 μM).

## Results and Discussion

**Scheme 1:**
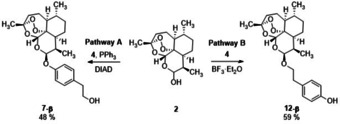
Stereoselective synthesis of **7‐β** and **12‐β** hybrids. The stereochemistry of the reaction was controlled by the experimental conditions applied in the activation of the OH group at C‐10 in DHA **2**.

**Figure 2:**
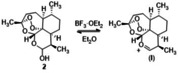
Formation of the planar oxacarbenium ion intermediate (I) from dihydroartemisin **2** in the presence of BF_3_ ⋅ Et_2_O. The approach of the nucleophile from the *Re*‐(β)‐face of (I) is favoured with respect to the *Si*‐(α)‐face due to the steric hindrance of the polycyclic part of the molecule.

Initially we developed a stereoselective synthesis of the DHA hybrid **5**, that was previously obtained as a diastereomeric mixture (1 : 1 ratio) of epimers at C‐10 position (numbering of molecule is reported in Figure 1). The stereoselectivity in the preparation of C‐10 ether derivatives of DHA usually depends on the nature of the coupling reagents, the Mitsunobu procedure affording the β‐epimer as exclusive or largely predominant product.^[16]^ On the basis of these data, DHA **2** (0.45 mmol) was treated with **4** (0.45 mmol) in the presence of PPh_3_ (0.45 mmol) and DIAD (0.45 mmol) in toluene (5 mL) and DMF (500 μL) at 25 °C^[17]^ to afford the epimer **7‐β** as the only recovered product in 48 % yield, besides to unreacted substrate (30 %) (Scheme 1, pathway A). In accordance with the expected stereoselective mechanism of Mitsunobu procedure, the β‐epimer of dihydroartemisinin was recovered as the only unreacted substrate. The stereochemistry of **7‐β** was confirmed by the NMR coupling constant between H‐10 and H‐9 [*J*_(H9, H10)_=3.2 Hz], corresponding to the cis‐configuration of the adjacent protons. This value is of the same order of magnitude than that of other β‐epimers of DHA.^[18]^ The high stereoselectivity of the reaction was probably due to steric hindrance of the β‐methyl group at C‐9^[19]^ favoring barrierless formation of the C‐10α‐PPh_3_/hemiacetal hydroxy adduct, followed by nucleophilic displacement from tyrosol. As an alternative, treatment of **2** (0.37 mmol) with **4** (0.37 mmol) in the presence of BF_3_ ⋅ Et_2_O (0.37 mmol) in Et_2_O (13 mL) at 0 °C afforded epimer **12‐β** (Scheme 1, pathway B), beside to the β‐methyl glycal anhydrodihydroartemisinin (not shown), derived from the skeletal rearrangement for the neutralization of the oxacarbenium ion intermediate (I, Figure 2). In this compound the coupling constant between H‐9 and H‐10 (3.6 Hz), confirmed the cis‐diaxial configuration of the pyranose ring.^[20]^ Previous data dealing with the role of BF_3_ ⋅ Et_2_O in the formation of a planar oxacarbenium ion intermediate (**I**) (Figure 2) followed by the preferential attack of the nucleophile from the *Re*‐(β)‐face of the molecule are reported.^[21]^ In addition, the conversion of DHA β‐epimers to corresponding α‐counterparts in BF_3_ ⋅ Et_2_O is a thermodynamically favored process.^[22]^ The nucleophilic addition of tyrosol on intermediate (**I**) was regiospecific due to the higher nucleophile character of the primary aliphatic alcohol with respect to the phenolic counterpart.^[23]^

**Scheme 2:**
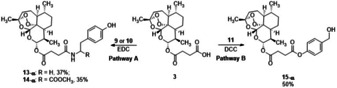
Synthesis of hybrids **13‐α**, **14‐α** and **15‐α** by EDC, or in alternative, DCC mediated esterification procedures. The reactions were performed starting from the α‐epimer of ART. The original stereochemistry at C‐10 was retained in the reaction products.

Successively, three novel ART hybrids were synthesized by the use of tyramine **9**, L‐tyrosine methyl ester **10** and 4‐hydroxybenzyl alcohol **11** in order to realize a spacer morphing study. Tyramine differs from tyrosol for the amino group instead of the primary hydroxyl moiety, while tyrosine methyl ester is characterized by the α‐carbon functionalization of tyramine. 4‐hydroxybenzyl alcohol is an inferior homolog of tyrosol. Briefly, the treatment of **3** with equimolar amount of **9** or **10**, in the presence of EDC ⋅ HCl (0.25 mmol) and HOBt (0.25 mmol), in DMF (2 mL) at 25 °C afforded ART hybrids **13‐α** and **14‐α** in 37 % and 35 % yield, respectively, besides to artesunic acid **3** (Scheme 2, pathway A). The novel hybrids retained the original chirality at C‐10 as confirmed by the NMR *J*_(H9, H10)_ coupling constant. The reaction proceeded with high regiospecificity to afford the corresponding amide derivatives. In addition, **15‐α** was obtained in 50 % yield by reaction of **3** (0.66 mmol) with **11** (0.30 mmol) under Steglich esterification condition,^[24]^ involving the use of DCC (0.30 mmol) and DMAP (0.44 mmol) in CH_2_Cl_2_ (2.5 mL) at 25 °C (Scheme 2, pathway B).

**Scheme 3:**
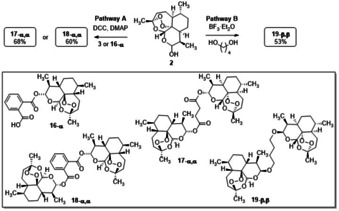
Synthesis of dimers **17‐α**,**α**, **18‐α**,**α** and **19‐β**,**β** from dihydroartemisinin **2**.

A panel of novel six DHA and ART dimers was then prepared. In a first set of experiments two dimers were obtained by reaction of DHA **2** with ART **3** or, in alternative, with **16‐α** (prepared as reported in Ref. 25) (Scheme 3).

As a general procedure, **2** (0.52 mmol) was treated with equimolar amount of **3** or **16‐α**, DCC (0.52 mmol) and DMAP (0.16 mmol) in CH_2_Cl_2_ (3 mL) at 25 °C to afford dimers **17‐α**,**α** and **18‐α**,**α** in 68 % and 60 % yields, respectively (Scheme 3, pathway A). Conversely, the dimer **19‐β**,**β** (53 % yield) was prepared by reaction of **2** (1.0 mmol) with 1,4‐butandiol (0.5 mmol) and BF_3_ ⋅ Et_2_O (1.0 mmol) in Et_2_O (30 mL) at 0 °C (Scheme 3, pathway B). In this latter case, **19‐β**,**β** was selectively obtained from the oxacarbenium ion intermediate (**I**) by thermodynamically driven equilibration of the epimers.[^16^, ^22^]

**Scheme 4:**
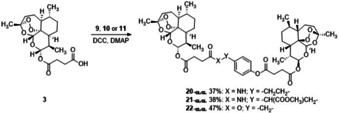
Synthesis of dimers **20‐α**,**α**, **21‐α**,**α** and **22‐α**,**α** from artesunic acid **3**.

Three further dimers were synthesized by reaction of **3** (1.0 mmol) with compounds **9**, **10**, and **11** (0.5 mmol) in the presence of DCC (1.1 mmol) and DMAP (0.3 mmol) in CH_2_Cl_2_ (4 mL) at room temperature to afford compounds **20**–**22** with appreciable yield (37 %, 38 % and 47 %, respectively) (Scheme 4). The α,α‐configuration of the C‐10 position was retained as determined by NMR *J*_(H9,H10)_ coupling constants.

The stability of the novel synthesized derivatives was evaluated according to Tsogoeva *et al* ^[26]^ by heating the DHA and ART hybrids and dimers at 60 °C for 24 h. ^1^H NMR registered less than 5 % of decomposition, confirming the stability of novel derivatives. Compound **22‐α**,**α** was evaluated after 24 and 48 hours (25 °C, pH=7) of exposition to assay medium by the use of High‐Performance Liquid Chromatography (HPLC) in comparison with artesunic acid **3**, demonstrating a good stability (Figures S#1‐5).

The anticancer‐activity of DHA and ART hybrids **7‐β**, **12‐β**, **13‐α**, **14‐α**, **15‐α**, and **16‐α**, and of DHA and ART dimers **17‐α**,**α**, **18‐α**,**α**, **19‐β**,**β**, **20‐α**,**α**, **21‐α**,**α** and **22‐α**,**α**, was evaluated by the cell survival MTT assay on metastatic melanoma cancer cell line RPMI7951. Artemisinin **1**, DHA **2**, ART **3**, hybrids **5**–**7**, dimer **8**, and commercially available drug paclitaxel (Taxol) were used as reference. In addition, data were compared with experiments performed on normal human primary fibroblast cell line (C3PV). Table 1 reports the IC_50_ (half‐maximal inhibitory concentration) and CC_50_ (half‐maximal cytotoxic concentration) values of the tested compounds.

**Table 1:** Biological activity of novel DHA and ART hybrid and dimer derivatives against metastatic melanoma cancer cell lines RPMI7951.^[a]^

Entry	Type	Compound	CC_50_±SD^[b]^ C3PV	IC_50_±SD^[c]^ RPMI7951
1	–	1	>300.0±14.85	3.62±0.99
2	–	2	0.68±0.19	0.91±0.45
3	–	3	1.68±0.44	1.08±0.56
4	DHA Hybrid	5^[d]^	1.76±0.31	0.33±0.08
5	ART Hybrid	6^[d]^	>300.0±10.71	8.34±3.06
6	ART Hybrid	7^[d]^	132±12.58	0.09±0.03
7	ART DIMER	8^[d]^	6.10±3.74	0.49±0.05
8	DHA Hybrid	7‐β	1.14±0.45	0.2±0.01
9	DHA Hybrid	12‐β	38.15±2.56	0.4±0.03
10	ART Hybrid	13‐α	3.39±0.05	1.7±0.03
11	ART Hybrid	14‐α	6.38±1.5	1.3±0.07
12	ART Hybrid	15‐α	55.25±7.33	2.5±0.05
13	DHA Hybrid	16‐α	8.14±0.85	4.6±0.03
14	DHA DIMER	17‐α,α	0.25±0.03	0.07±0.01
15	DHA DIMER	18‐α,α	8.0±0.1	10.75±1.6
16	DHA DIMER	19‐β,β	5.7±0.79	3.6±0.95
17	ART DIMER	20‐α,α	228.0±1.5	2.45±0.05
18	ART DIMER	21‐α,α	>300.0±7.56	1.76±0.03
19	ART DIMER	22‐α,α	>300.0±9.78	0.05±0.02
20	–	Paclitaxel	78.88±0.79	0.013±0.10

^[a]^All experiments were conducted in triplicate. ^[b]^CC_50_±SD (half‐maximal cytotoxic concentration±standard deviation) values for all compounds are expressed in micromolar units. ^[c]^IC_50_±SD (half‐maximal inhibitory concentration±standard deviation) values for all compounds are expressed in micromolar units. ^[d]^Antimelanoma and cytotoxicity data from ref. 12.

Hybrid and dimer derivatives showed antimelanoma activity in the micromolar/nanomolar range (10.75–0.05 μM), the ART dimers **20‐α**,**α**, **21‐α**,**α** and **22‐α**,**α** being characterized by low cytotoxicity. The regiospecific linkage of the alcohol moiety of tyrosol did not affect neither the biological activity nor the cytotoxicity of products, as highlighted by the comparison of the IC_50_ value of **7‐β** versus **12‐β** (Table 1, entry 8 versus entry 9). In addition, the antimelanoma activity of **12‐β** was of the same order of magnitude than racemic derivative **5** (Table 1, entry 4 versus entry 9). On the basis of these data the stereochemistry of C‐10 was not relevant for the biological activity of hybrid **12**. Hybrids **13‐α**, **14‐α**, and **15‐α**, showed a significative antimelanoma effect associated to a pronounced cytotoxicity, less pronounced in the case of compound **15‐α** (Table 1, entries 10–12). Dimers **17‐α**,**α**, **18‐α**,**α** and **19‐β**,**β** showed interesting antimelanoma activity, especially in the case of compound **17‐α**,**α** (Table 1, entry 14), unfortunately accompanied by strong cytotoxicity. In this latter case, the substitution of the succinic acid spacer in **17‐α**,**α** with a more rigid (compound **18‐α**,**α**), or highly flexible (compound **19‐β**,**β**) linker did not increase the antimelanoma activity (Table 1, entries 15 and 16). With respect to DHA hybrids, the presence of a second DHA scaffold slightly decreased the antimelanoma effect, as showed by the comparison between dimer **18‐α**,**α** and hybrid **16‐α** (Table 1, entry 13 versus entry 15). These two derivatives showed a lower activity compared to the parent compound DHA **2**, further suggesting the detrimental role of the rigid counterpart/spacer phthalic acid in the antimelanoma efficacy. Finally, ART dimers **20‐α**,**α**, **21‐α**,**α** and **22‐α**,**α** performed as the best products of the series showing low toxicity and high antimelanoma activity (Table 1, entries 17–19). In particular, compound **22‐α**,**α** bearing the 4‐hydroxybenzyl alcohol spacer, was characterized by antimelanoma activity two orders of magnitude higher than that of the parent artesunic acid (Table 1, entry 3 versus entry 19), and of the same order of magnitude than commercial drug paclitaxel (Table 1, entry 19 versus entry 20).

It is noteworthy that the substitution of the tyrosol spacer with a molecular framework containing nitrogen, or alternatively with a smaller side chain, reduced significantly the toxicity of the product, contemporary retaining a high value of antimelanoma activity (Table 1, entry 7 versus entries 17–19). The effect of dimers **20**–**22‐α**,**α** was further evaluated against other tumour types, such as human prostate and breast cancers in PC3 and MDA‐MB‐231 cell lines, respectively (Table 2).

**Table 2:** Biological activity of dimers **20**–**22‐α**,**α** against metastatic melanoma cancer cell lines RPMI7951 in presence and absence of DFO, and against human prostate (PC3) and breast (MDA‐MB‐231) cancer cell lines.^[a]^

Entry	Dimer	IC_50_±SD^[b]^			
		PC3	MDA‐ MB‐231	RPMI7951^[c]^	RPMI7951‐ DFO^[d]^
**1**	**20‐α**,**α**	2.3±0.35	1.3±0.85	2.45±0.05	2.90±0.06
**2**	**21‐α**,**α**	3.04±0.34	3.08±0.85	1.76±0.03	5.11±0.45
**3**	**22‐α**,**α**	3.1±0.19	2.4±0.95	0.05±0.02	0.88±0.02

^[a]^All experiments were conducted in triplicate. ^[b]^IC_50_±SD (half‐maximal inhibitory concentration±standard deviation) values for all compounds are expressed in micromolar units. ^[c]^Experiment conducted in absence of DFO. ^[d]^Experiment conducted in presence of DFO.

As depicted in the Table 2, **20‐α**,**α** and **21‐α**,**α** were active against prostate and breast cancers in the micromolar range, as in the case of RPMI7951. On the contrary, dimer **22‐α**,**α** turned out to be two order of magnitude less potent on PC3 and MDA‐MB‐231 compared to RPMI7951, demonstrating a cancer‐type selectivity for metastatic melanoma. Cell viability assay on RPMI7951 cell line of **20**–**22‐α**,**α** was also repeated in the presence of iron chelating agent DFO to evaluate a possible role of this metal in the biological activity. As reported in Table 2, the presence of DFO decreased the activity of **21‐α**,**α** (entry 2), with a more pronounced effect for **22‐α**,**α** (entry 3). These results suggest that the antimelanoma effect for dimer **22‐α**,**α** could be due to the presence of iron triggered radical cascade mechanisms with subsequent endoperoxide ring‐opening.

**Figure 3:**
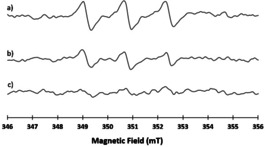
X‐band EPR spectra of the reaction of a) **20‐α**,**α**, b) **22‐α**,**α**, c) **21‐α**,**α** in the presence of MNP at t=150 minutes after the addition of the last reagent Fe(II)SO_4_ *. Experimental condition*. 9.866 GHz microwave frequency, 0.1 mT modulation amplitude and 0.2 mW microwave power.

EPR experiments in the presence of Fe(II)SO_4_ and the spin trap MNP [0, 15, 70, 120, 150 and 180 min with respect to the addition of the last reagent Fe(II)SO_4_] were also performed. Spectra were recorded till 180 minutes after the addition of Fe(II)SO_4_ to **20**–**22‐α**,**α** samples and compared with reference obtained by adding only MNP (Figures S#6–8). In Figure 3 a comparison of the EPR spectra of the MNP adduct from **20**–**22‐α**,**α** at t=150 minutes is reported in order to compare the intensity of the radical formation for the three products at the same time. In all cases the g_iso_=2.0063±0.0001 and the coupling constant of nitrogen was A=1.62±0.01 mT. These magnetic parameters are in agreement to a C‐centered radical as previously published.^[12]^ In particular, in the case of **20‐α**,**α** the radical signal was observed after 1 hour, then increased in intensity and remained stable till 150 minutes [Figures S#6 and 3a)].

For compound **22‐α**,**α** the signal is visible at 120 minutes, reached its maximum at 150 minutes and was still present at 180 minutes (the intensity of the radical was the highest among all the three cases) [Figures S#8 and 3b)]. On the contrary, in **21‐α**,**α** the radical signal was almost undetectable even after 180 minutes [Figures S#7 and 3c)].

Results obtained for **22‐α**,**α** further confirm the beneficial role of iron for the mechanism of action of this dimer and the possible correlation of its anticancer activity with the formation of C‐centered radicals.

## Conclusion

A library of 11 novel derivatives of artemisinin and artesunic acid with hybrid and dimer structure was obtained by the use of stereoselective experimental conditions. The novel products were evaluated for their cancer selectivity by cell survival MTT assay against metastatic melanoma cancer cell line RPMI7951, using normal human primary fibroblast C3PV as a reference. The artesunate dimers 20‐α,α, 21‐α,α, and 22‐α,α emerged as the most active and low toxic derivatives of the series, highlighting the importance of the artesunic acid scaffold in the biological activity. In particular, compound 22‐α,α, showed an IC_50_ comparable with the antimelanoma approved drug paclitaxel, and significantly higher than the parent compound artesunic acid (0.05 vs 1.08 μM). DFO assays and EPR analysis let to hypothesize a correlation between the biological effect and the formation of an iron dependent C‐centered radical intermediate. In addition, cancer selectivity experiments conducted on prostate and breast tumor cell lines showed a high selectivity of **22‐α,α** toward metastatic melanoma cell lines. Regarding spacer morphing study, tyramine, L‐tyrosine methyl ester and 4‐hydroxybenzyl alcohol performed as spacer frameworks better than previously studied tyrosol moiety, affording compounds characterized by lower toxicity and high antimelanoma activity.

## Experimental Section

### Cell culture condition

The primary human fibroblast C3PV cell line was treated according to Botta et al.^[27]^ Metastatic melanoma cell line (RPMI7951) was grown in Eagle's Minimum Essential Medium (EMEM) containing 15 % and 10 % FBS respectively, in addition to penicillin (100 U/ml) and streptomycin (1 mg/ml). The cell lines were maintained at 37 °C in a humidified atmosphere (95 %) in the presence of 5.0 % CO_2_. Prostate cancer (PC3) and breast cancer (MDA‐MB‐231) cell lines were raised in DMEM/F12 and RMPI1640 medium, respectively. To the medium was added 10 % FBS, 1 mM Glutammine and 40 μg/ml of Gentamicin. All cell lines were mantained at 37 °C in a humidified atmosphere (95 %) in the presence of 5.0 of CO_2_.

### Treatment Protocol

To study the effect of artemisinin and its derivatives on cell viability C3PV, RPMI7951, PC3, MDA‐MB‐231 cell lines were seeded in 96‐well plates (6000 cells/well in 100 μl medium) and incubated overnight to allow cell adherence. After, the medium was replaced with fresh medium containing the appropriate dose of compound. Artemisinin and its derivatives were used in a range of 0.01 to 1.0 μM for 24 h. The analyses of cell viability were done at the end of treatment. The assays were performed in quadruplicate for both treatments.

### Statistical analysis

The CC_50_ and IC_50_ values were determined by non‐linear regression using the program graphpad prism 6. The results showed in Table 1 (in the main text) are expressed as the average of all experiments ± standard error.

### Cell viability assay

Cell viability was evaluated using MTT cell proliferation assay. Briefly, after incubation for 3 h at 37 °C with MTT (0.5 mg/ml) the supernatant was replaced with 100 μl of a lysis solution containing 10 % SDS, 0.6 % Acetic acid in DMSO to dissolve the formazan crystals. Optical density measurements were performed with a scanning spectrophotometer DTX880 Multimode Detector (Beckman Coulter) using a 630 nm (background) and a 570 nm filter.

### Treatment Protocol for DFO Assay

To study the mechanism of action of compounds **20**–**22α,α**, the SK‐MEL3, SK‐MEL24 and RPMI‐ 7951 cell lines were seeded in 96‐well plates (6000 cells/well in 100 μL of medium) and incubated overnight to allow cell adherence. Afterward, the medium was replaced with fresh medium containing DFO (20 μM) for 1 h. Then the appropriate dose of compounds **20–22α,α** were added for 24 h. The analyses of cell viability were done at the end of treatment. The assays were performed in quadruplicate for both treatments.

### Chemistry

*Procedure for the synthesis of derivative **7β***. PPh_3_ (120 mg, 0.45 mmol, 1 equiv.) and DIAD (89 μL, 0.45 mmol, 1 equiv.) were added to a cold (0 °C) stirred solution of dihydroartemisinin **2** (130 mg, 0.45 mmol, 1 equiv.) and tyrosol **4** (63 mg, 0.45 mmol, 1 equiv.) in a mixture of toluene (5 mL) and DMF (500 μL). The reaction mixture was stirred overnight at room temperature. The solvent was reduced under vacuum, then aqueous solution of lithium chloride 3 % (10 mL) was added and extracted with EtOAc (3×10 mL). The combined organic layers were washed with brine (10 mL), dried over Na_2_SO_4_, filtered and concentrated under reduced pressure. The crude was purified by flash column chromatography (EtOAc/Hex 3 : 1). Yield=48 %. R_*f*_=0.19 (EtOAc/Hex 3 : 2, molybdato phosphate). ^1^H‐NMR (CDCl_3_, 400 MHz): *δ*=7.16 (d, 2H, *J*=8.4 Hz), 7.08 (d, 2H, *J*=8.4 Hz), 5.52 (s, 1H), 5.50 (d, 1H, *J*=3.2 Hz), 3.84 (bt, 1H), 2.85–2.78 (m, 3H), 2.45‐2.37 (m, 1H), 2.07‐1.89 (m, 3H), 1.71–1.60 (m, 1H), 1.59‐1.37 (m, 3H), 1.35 (s, 3H), 1.30–1.27 (m, 2H), 1.13–1.05 (m, 1H), 1.01 (d, 3H, *J*=6.7 Hz), 0.98 (d, 3H, *J*=7.4 Hz) ppm. ^13^C‐NMR (CDCl_3_, 100 MHz): *δ*=156.2, 131.7, 130.0, 116.9, 104.2, 100.6, 88.2, 81.0, 63.8, 60.4, 52.5, 44.4, 37.1, 36.4, 34.7, 32.8, 30.2, 24.7, 24.5, 22.8, 14.2. MS (ESI): *m/z* for [C_23_H_33_O_6_]^+^=405. Anal. calcd. for C_23_H_32_O_6_: C, 68.29; H, 7.97; O, 23.73; found: C, 68.27; H, 7.96; O, 23.76.

*Procedure for the synthesis of derivative **12β***. To a stirred solution of dihydroartemisinin **2** (106 mg, 0.37 mmol, 1 equiv.) and tyrosol **4** (67.0 mg, 0.37 mmol, 1 equiv.) in dry diethyl ether (13 mL), BF_3_ ⋅ Et_2_O (205 μL, 0.37 mmol, 1 equiv.) was added at 0 °C. The stirring at 0 °C was continued for 60 minutes and then the reaction was quenched by addition of saturated solution of NaHCO_3_ (8 mL). After phase separation, the aqueous layer was extracted with Et_2_O (3×10 mL), washed with brine (10 mL), dried over Na_2_SO_4_, filtered and concentrated under reduced pressure. The crude product was purified by flash column chromatography (Hex/EtOAc 3 : 1). Yield=59 %. R_*f*_=0.27 (Hex/EtOAc 3 : 1, molybdato phosphate). ^1^H‐NMR (CDCl_3_, 400 MHz): *δ*=7.08 (d, 2H, *J*=8.4 Hz), 6.78‐6.76 (dd, 2H, *J*=6.4, 2.0 Hz), 5.15 (s, 1H), 4.79 (d, 1H, *J*=3.6 Hz), 4.06–4.03 (m, 1H), 3.59–3.56 (m, 1H), 2.83–2.79 (m, 2H), 2.61–2.57 (m, 1H), 2.37–2.35 (m, 1H), 2.04–2.94 (m, 1H), 1.88–1.83 (m, 1H), 1.67–1.53 (m, 2H), 1.46–1.40 (m, 1H), 1.39–1.27 (m, 2H), 1.31 (s, 3H) 1.20–1.15 (m, 2H), 0.99–0.85 (m, 2H), 0.95 (d, 3H, *J*=6 Hz), 0.87 (d, 3H, *J*=6 Hz) ppm. ^13^C‐NMR (CDCl_3_, 100 MHz): *δ*=154.0, 131.5, 130.1, 115.0, 104.0, 101.7, 87.8, 81.1, 69.0, 52.5, 44.3, 37.2, 36.4, 35.4, 34.6, 30.9, 26.2, 24.7, 24.3, 20.3, 13.0 ppm. MS (ESI): *m/z* for [C_23_H_33_O_6_]^+^=405. Anal. calcd. for C_23_H_32_O_6_: C, 68.29; H, 7.97; O, 23.73; found: C, 68.27; H, 7.96; O, 23.76

*General procedure for the synthesis of derivatives **13α** and **14α***. A solution of artesunic acid **3** (94.5 mg, 0.25 mmol, 1 equiv.) and HOBt (33.7 mg, 0.25 mmol, 1 equiv.) in dry DMF (2 mL) was cooled to 0 °C. EDC^.^ HCl (47.3 mg 0.25 mmol, 1 equiv.) was added at 0 °C under N_2_. After stirring the reaction mixture for 10 minutes, a solution of the opportune amine (tyramine **9** or tyrosine methylester **10**; 1 equiv.) and DIPEA (40.2 μL, 0.25 mmol, 1 equiv.) in dry DMF (2 mL) was added at 0 °C. The resulting mixture was slowly warmed to room temperature and stirred overnight. After this time, EtOAc (10 mL) and aqueous solution of lithium chloride 3 % (10 mL) were added. The two phases were separated, and the water phase was extracted with EtOAc (2×15 mL). The combined organic layers were washed with H_2_O (3×15 mL) and brine (20 mL), dried over Na_2_SO_4_ filtered and concentrated under reduced pressure. The residue was purified by flash column chromatography (DCM/MeOH 9.5 : 0.5).

**13α**: yield=37 %. R_*f*_=0.34 (DCM/MeOH 9 : 1, molybdato phosphate). ^1^H‐NMR (CDCl_3_, 400 MHz): *δ*=7.03 (d, 2H, *J*=8.0 Hz), 6.81 (d, 2H, *J*=8.0 Hz), 6.35 (s, 1H), 5.82 (s, 1H), 5.79 (d, 1H, *J*=12.0 Hz), 5.46 (s, 1H), 3.50–3.46 (m, 2H), 2.78–2.69 (m, 4H), 2.63–2.57 (m, 1H), 2.49–2.36 (m, 3H), 2.06–2.03 (m, 1H), 1.92–1.80 (m, 3H), 1.72–1.65 (m, 1H), 1.62–1.47 (m, 2H), 1.44 (s, 3H), 1.27–1.07 (m, 2H), 1.09–1.00 (m, 1H), 0.99 (d, 3H, *J*=8.0 Hz), 0.87(d, 3H, *J*=8.0 Hz) ppm. ^13^C‐NMR (CDCl_3_, 100 MHz): *δ*=171.8, 171.4, 154.9, 130.3, 129.8, 115.6, 104.6, 92.3, 91.6, 80.2, 51.6, 45.2, 40.9, 37.3, 36.2, 34.7, 34.1, 31.8, 30.9, 29.8, 25.9, 24.6, 21.9, 20.2, 12.0 ppm. MS (ESI): *m/z* for [C_27_H_38_NO_8_]^+^=504. Anal. calcd. for C_27_H_37_O_8_: C, 64.40; H, 7.41; N, 2.78; N, 2.78; O, 25.42; found: C, 64.38; H,7.40; N, 2.79; O, 25.43

**14α**: yield=35 %. R_*f*_=0.41 (DCM/MeOH 9 : 1, molybdato phosphate). [α]_D_=+12.56 (c 1.0, CHCl_3_); ^1^H‐NMR (CDCl_3_, 400 MHz): *δ*=8.63 (s, 1H), 6.96 (d, 2H, *J*=8.4 Hz), 6.78 (d, 2H, *J*=8.4 Hz), 6.20 (d, 1H, *J*=8.0 Hz), 5.79 (d, 2H, *J*=9.6 Hz), 5.46 (s, 1H), 4.84–4.82 (m, 1H), 3.72 (s, 3H), 3.10–3.03 (m, 2H), 2.76–2.52 (m, 4H), 2.51–2.48 (m, 1H), 2.47–2.38 (m, 1H), 2.06–2.02 (m, 1H), 1.92–1.90 (m, 1H), 1.79–1.70 (m, 2H), 1.64–1.60 (m, 1H), 1.40 (s, 3H), 1.37–1.27 (m, 1H), 1.10–1.04 (m, 1H), 1.03 (d, 3H, *J*=6.8 Hz), 0.86 (d, 3H, *J*=6.8 Hz) ppm. ^13^C‐NMR (CDCl_3_, 100 MHz): *δ*=172.0, 171.6, 170.9, 155.5, 149.4, 136.4, 130.3, 127.0, 123.9, 115.6, 104.5, 92.2, 91.5, 80.1, 53.3, 52.3, 51.5, 45.2, 37.2, 36.2, 34.0, 31.7, 30.4, 29.3, 25.8, 24.5, 21.9, 20.1, 12.0. ppm. MS (ESI): *m/z* for [C_29_H_40_NO_10_]^+^=562. Anal. calcd. for C_29_H_39_O_10_: C, 62.02; H, 7.00; N, 2.49; O, 28.49; found: C, 62.04; H, 7.01; N, 2.47; O, 28.47

*Procedure for the synthesis of derivative **15α***. To a solution of artesunic acid **3** (253 mg, 0.66 mmol, 1 equiv.) in dry CH_2_Cl_2_ (2.5 mL), DCC (326.30 mg, 0.30 mmol, 1 equiv.) and DMAP (54.75 mg, 0.44 mmol, 0.68 equiv.) were added at room temperature. After the addition of 4‐hydroxybenzyl alcohol **11** (37. 24 mg, 0.30 mmol, 1 equiv.), the reaction mixture was stirred overnight under N_2_ atmosphere. The precipitated dicyclohexylurea was removed by filtration and the solvent was removed under reduced pressure. The residue was purified by flash column chromatography (Hex/EtOAc 1.5 : 1). Yield=50 %. R_*f*_=0.18 (Hex/EtOAc 4 : 1, molybdato phosphate). ^1^H‐NMR (CDCl_3_, 400 MHz): *δ*=7.39 (d, 2H, *J*=8.4 Hz), 7.12 (d, 2H, *J*=8.4 Hz), 5.85 (d, 1H, *J*=10.0 Hz), 5.47 (s, 1H), 4.70 (s, 2H), 3.47–3.51 (m, 1H), 2.89–2.85 (m, 4H), 2.62–2.57 (m, 1H), 2.44–2.36 (m, 1H), 2.06–2.01 (m, 1H), 1.95–1.91(m, 2H), 1.76–1.71 (m, 2H), 1.63–1.61 (m, 1H), 1.45 (s, 3H), 1.38–1.25 (m, 1H), 1.16–1.01 (m, 1H), 0.99 (d, 3H, *J*=6 Hz), 0.87 (d, 3H, *J*=6.8 Hz) ppm. ^13^C‐NMR (CDCl_3_, 100 MHz): *δ*=171.3, 171.2, 149.5, 140.5, 127.9, 121.7, 104.0, 92.4, 91.1, 80.3, 79.6, 62.8, 51.5, 45.2, 36.4, 34.1, 33.8, 32.1, 31.1, 29.2, 25.9, 25.7, 24.6, 21.4, 20.5, 12.2 ppm. MS (ESI): *m/z* for [C_26_H_35_O_9_]^+^=491. Anal. calcd. for C_26_H_34_O_9_: C, 63.66; H, 6.99 O, 29.35; found: C, 63.67; H, 6.98; O, 29.37

*Procedure for the synthesis of derivative **17α,α***. To a solution of artesunic acid **3** (1.0 equiv.) in dry DCM (3.0 mL), DCC (107 mg, 0.52 mmol, 1.0 equiv.) and DMAP (61 mg, 0.16 mmol, 0.3 equiv.) were added at room temperature. After the addition of dihydroartemisinin **2** (147 mg, 0.52 mmol, 1.0 equiv.), the reaction mixture was slowly stirred overnight under N_2_ atmosphere. The precipitated dicyclohexylurea was removed by filtration and the solvent was removed under reduced pressure. The residue was purified by flash column chromatography (Hex/EtOAc 1 : 1). Yield=68 %. R_*f*_=0.27 (Hex/EtOAc 1 : 1, molybdato phosphate). ^1^H‐NMR (CDCl_3_, 400 MHz): *δ*=5.80 (d, 2H, *J*=10.0 Hz), 5.44 (s, 2H), 2.84–2.79 (m, 8H), 2.68–2.58 (m, 2H), 2.42–2.34 (m, 2H), 1.90–1.88 (m, 2H), 1.79–1.71 (m, 4H), 1.65–1.61 (m, 2H), 1.54–1.52 (m, 2H), 1.51 (s, 6H), 1.39–1.29 (m, 2H), 1.27–1.22 (m, 2H), 1.0‐6‐1.03 (m, 2H), 0.97 (d, 6H, *J*=5.6 Hz), 0.88 (d, 6H, *J*=5.6 Hz) ppm. ^13^C‐NMR (CDCl_3_, 100 MHz): *δ*=171.0, 104.4, 92.1, 91.4, 80.1, 51.5, 45.2, 37.2, 36.2, 34.0, 31.7, 28.8, 25.9, 24.5, 22.0, 20.2, 12.0 ppm. MS (ESI): *m/z* for [C_34_H_51_O_12_]^+^=651. Anal. calcd. for C_34_H_50_O_12_: C, 62.75; H, 7.74 O, 29.50; found: C, 62.73; H, 7.72; O, 29.51

*Procedure for the synthesis of derivative **18α,α***. To a solution of compound **16α** (74 mg, 0.17 mmol, 1.0 equiv.) in dry CH_2_Cl_2_ (10 mL), DCC (43 mg, 0.20 mmol, 1.2 equiv.) and DMAP (7.22 mg, 0.05 mmol, 0.3 equiv.) were added and stirred for 15 minutes. After this period dihydroartemisinin **2** (60 mg, 0.21 mmol, 1.2 equiv.) was added to the mixture and the reaction was slowly stirred overnight under N_2_ atmosphere. The organic layer was filtered over celite, washed with HCl 1 M (10 mL) and brine (10 mL); dried over Na_2_SO_4_ and evaporated under vacuum. The crude product was purified by flash column chromatography (Hex/EtOAc 3 : 1). Yield=60 %. R_*f*_=0.27 (Hex/EtOAc 1 : 1, molybdato phosphate). [α]_D_=+22.21 (c 1.0, CHCl_3_); ^1^H‐NMR (CDCl_3_, 400 MHz): *δ*=7.88–7.86 (m, 2H) 7.58–7.55 (m, 2H), 5.98 (d, 2H, *J*=10.0), 5.50 (s, 2H), 2.70–2.67 (m, 2H), 2.43–2.39 (m, 2H), 2.06–2.03 (m, 2H), 1.86–1.83 (m, 4H), 1.77–1.63 (m, 2H), 1.49–1.48 (m, 2H), 1.45 (s, 6H), 1.34–1.32 (m, 4H), 1.21–1.06 (m, 4H), 1.02–0.92 (m, 12H), 0.92–0.85 (m, 2H) ppm. ^13^C‐NMR (CDCl_3_, 100 MHz): *δ*=165.9, 131.7, 131.1, 129.3, 104.3, 93.0, 91.5, 80.1, 51.6, 45.4, 37.3, 36.2, 34.1, 31.9, 29.7, 25.9, 24.6, 22.1, 12.2 ppm. MS (ESI): *m/z* for [C_38_H_51_O_12_]^+^=699. Anal. calcd. for C_38_H_50_O_12_: C, 65.31; H, 7.21 O, 27.47; found: C, 65.30; H, 7.20; O, 27.48

*Procedure for the synthesis of derivative **19β,β***. To a stirred solution of dihydroartemisinin **2** (286 mg, 1.0 mmol, 1 equiv.) in Et_2_O (5 mL) was added dry 1,4‐butanediol (44 μL, 0.5 mmol, 0.5 equiv.) and BF_3_ ⋅ Et_2_O (59.9 μL, 0.70 mmol, 1 equiv.) at 0 °C. The stirring at 0 °C was continued for 90 minutes and then the reaction was quenched by addition of saturated solution of NaHCO_3_ (8 mL). After phase separation, the aqueous layer was extracted with Et_2_O (3×10 mL), washed with brine (10 mL), dried over Na_2_SO_4_, filtered and concentrated under reduced pressure. The crude product was purified by flash column chromatography (Hex/EtOAc 5 : 1). Yield=53 %. R_*f*_=0.27 (Hex/EtOAc 9 : 1, molybdato phosphate). ^1^H‐NMR (CDCl_3_, 400 MHz): *δ*=5.39 (s, 2H), 4.78 (d, 2H, *J*=3.4 Hz), 3.90–3.87 (m, 2H), 3.68–3.65 (m, 4H), 3.43–3.40 (m, 2H), 2.64–2.62 (m, 2H), 2.37–2.34 (m, 2H), 2.06–2.02 (m, 2H), 1.90–1.81 (m, 2H), 1.78–1.62 (m, 6H), 1.52–1.46 (m, 2H), 1.44 (s, 6H), 1.33‐1.23 (m, 8H), 0.96 (d, 6H, *J*=6.0 Hz), 0.91 (d, 6H, *J*=7.2 Hz) ppm. ^13^C‐NMR (CDCl_3_, 100 MHz): *δ*=104.1, 102.0, 87.9, 81.1, 68.2, 62.6, 52.5, 44.4, 37.4, 36.4, 34.6, 30.9, 29.7, 26.1, 24.6, 24.4, 20.3, 12.9 ppm. MS (ESI): *m/z* for [C_34_H_55_O_10_]^+^=623. Anal. calcd. for C_34_H_54_O_10_: C, 65.57; H, 8.74; O, 25.69; found: C, 65.60; H, 8.71; O, 25.70

*General procedure for the synthesis of derivatives **20α,α**, **21α,α** and **22α,α***. To a solution of artesunic acid **3** (384 mg, 1 mmol, 2 equiv.) in dry CH_2_Cl_2_ (10 mL), DCC (227 mg, 1.1 mmol, 2.2 equiv.) and DMAP (36.6 mg, 0.3 mmol, 0.6 equiv.) were added and the mixture was stirred for 15 minutes. After this period the opportune spacer (tyramine **9**, tyrosine methylester **10**, or alcohol, 4‐hydroxybenzyl alcohol **11**; 1 equiv.) was added and the reaction was stirred overnight under N_2_ atmosphere. The organic layer was filtered over celite, washed with HCl 1 M (10 mL) and brine (10 mL); dried over Na_2_SO_4_ and evaporated under vacuum. The crude product was purified by flash column chromatography (Hex/EtOAc 1.5 : 1).

**20α,α**: yield=37 %. R_*f*_=0.50 (Hex/EtOAc 1.5 : 1, molybdato phosphate). ^1^H‐NMR (CDCl_3_, 400 MHz): *δ*=7.21 (d, 2H, *J*=8.4 Hz), 7.06 (d, 2H, *J*=8.4 Hz), 5.83 (d, 1H, *J*=10.0 Hz), 5.79 (d, 1H, *J*=10.0 Hz), 5.70 (bt, 1H), 5.46 (s, 1H), 5.44 (s, 1H), 3.51–3.48 (m, 2H), 2.84–2.80 (m, 4H), 2.58–2.49 (m, 2H), 2.47–2.35 (m, 2H), 2.05–2.03 (m, 4H), 1.91–1.89 (m, 4H), 1.76–1.71 (m, 8H), 1.57–1.46 (m, 2H), 1.45 (s, 3H), 1.43 (s, 3H), 1.39–1.32 (m, 6H), 1.09‐1.01 (m, 2H), 0.98‐0.97 (m, 6H), 0.87–0.85 (m, 6H) ppm. ^13^C‐NMR (CDCl_3_, 100 MHz): *δ*=171.8, 171.7, 171.5, 171.0, 155.1, 149.0, 136.6, 130.0, 129.7, 121.6, 115.6, 104.6, 104.5, 92.2, 91.5, 80.1, 60.4, 51.5, 45.1, 41.1, 40.8, 37.3, 36.2, 35.0, 34.6, 34.0, 31.7, 31.6, 30.8, 29.7, 29.2, 29.0, 25.9, 25.8, 24.6, 22.6, 22.1, 21.1, 20.2, 14.2, 14.1, 12.0 ppm. MS (ESI): *m/z* for [C_46_H_64_NO_15_]^+^=870. Anal. calcd. for C_46_H_63_NO_15_: C, 63.51; H, 7.30; N, 1.61; O, 27.58; found: C, 63.52; H, 7.29; N, 1.59; O, 27.60.

**21α,α**: yield=38 %. R_*f*_=0.12 (Hex/EtOAc 1.5 : 1, molybdato phosphate). ^1^H‐NMR (CDCl_3_, 400 MHz): *δ*=7.13 (d, 2H, *J*=8.4 Hz), 7.04 (d, 2H, *J*=8.4 Hz), 6.07 (d, 1H, *J*=8.0 Hz), 5.83 (d, 1H, *J*=9.6 Hz), 5.80 (d, 1H, *J*=10.0 Hz), 5.46 (s, 1H), 5.45 (s, 1H), 4.90–4.85 (m, 1H), 3.71 (s, 3H), 3.12 (d, 2H, *J*=5.6 Hz), 2.86–2.84 (m, 8H), 2.58‐2.52 (m, 2H), 2.38–2.35 (m, 2H), 2.05–2.02 (m, 2H), 1.92–1.90 (m, 2H), 1.75–1.71 (m, 4H), 1.61–1.58 (m, 4H), 1.41 (s, 3H), 1.40 (s, 3H), 1.38–1.27 (m, 6H), 1.04–1.00 (m, 2H), 0.98–0.97(m, 6H), 0.87–0.85 (m, 6H) ppm. ^13^C‐NMR (CDCl_3_, 100 MHz): *δ*=171.7, 171.5, 170.9,170.7, 149.7, 133.4, 130.4, 130.3, 121.6, 115.5, 104.5, 92.3, 92.1, 91.5, 80.1, 53.4, 53.2, 53.1, 52.3, 51.5, 45.2, 37.3, 37.0, 36.2, 34.1, 33.9, 31.8, 30.5, 29.7, 29.3, 29.2, 29.1, 25.9, 25.5, 24.9, 24.6, 22.0, 21.0, 20.2, 14.2, 12.0 ppm. MS (ESI): *m/z* for [C_48_H_66_NO_17_]^+^=928. Anal. calcd. for C_48_H_65_NO_17_: C, 62,12; H, 7.06; N, 1.51; O, 29.31; found: C, 62.10; H, 7.05; N, 1.53; O, 29.32

**22α,α**: yield=47 %. R_*f*_=0.50 (Hex/EtOAc 9 : 1, molybdato phosphate). [α]_D_=+38.45 (c 1.0, CHCl_3_); ^1^H‐NMR (CDCl_3_, 400 MHz): *δ*=7.09 (d, 2H, *J*=8.6 Hz), 7.08 (d, 2H, *J*=8.6 Hz), 5.81 (d, 1H, *J*=9.6 Hz), 5.77 (d, 1H, *J*=9.6 Hz) 5,44 (s, 1H), 5.43 (s, 1H), 5.10 (s, 2H), 2.86–2.82 (m, 4H), 2.73–2.66 (m, 4H), 2.50–2.54 (m, 2H), 2.37–2.33 (m, 2H), 2.03–2.00 (m, 2H), 1.90–1.87 (m, 2H), 1.78–1.69 (m, 4H), 1.63–1.58 (m, 2H), 1.65–1.43 (m, 2H), 1.42 (s, 3H), 1.41 (s, 3H), 1.40–1.27 (m, 4H), 1.07–0.97 (m, 2H), 0.96–0.95 (m, 6H), 0.84 (d, 3H, *J*=7.2 Hz), 0.81 (d, 3H, *J*=7.2 Hz) ppm. ^13^C‐NMR (CDCl_3_, 100 MHz): *δ*=171.2, 171.1, 170.0, 170.6, 150.5, 134.9, 133.4, 129.4, 121.7, 108.8, 104.5, 103.7, 91.5, 91.4, 91.2, 89.7, 65.9, 60.4, 51.5, 51.4, 45.4, 45.2, 44.6, 43.8, 37.4, 37.2, 36.2, 34.1, 32.9, 31.8, 30.7, 29.2, 28.9, 25.9, 24.8, 24.6, 22.6, 22.0, 21.0, 20.3, 16.2, 14.1, 13.1, 12.06, 12.05 ppm. MS (ESI): *m/z* for [C_45_H_61_O_16_]^+^=858. Anal. calcd. for C_45_H_60_O_16_: C, 63.07; H, 7.06; O, 29.87; found: C, 63.05; H, 7.05; O, 29.90.

## Abbreviation

Triphenylphoshine (PPh_3_), Diisopropyl azodicarboxylate (DIAD), *N*,*N*‐dicyclohexylcarbodiimide (DCC); dimethylaminopyridine (DMAP); dichloromethane (CH_2_Cl_2_); chloroform (CHCl_3_); diethyl ether (Et_2_O); 1‐Hydroxybenzotriazole hydrate (HOBt); dimethylformamide (DMF); hexane (Hex); sodium bicarbonate (NaHCO_3_) *N*‐ethyl‐*N’*‐3 (dimethylaminopropyl)carbodiimidehydrocloride (EDC ⋅ HCl); Boron trifluoride (BF_3_); BF_3_‐diethyl etherate (BF_3_ ⋅ Et_2_O); Potassium carbonate (K_2_CO_3_); Ethyl acetate (EtOAc); 3‐(4,5‐dimethylthiazol‐2‐yl)‐2,5‐diphenyltetrazolium bromide (MTT); deferoxamine (DFO); Electron paramagnetic resonance (EPR); 2‐methyl‐nitrosopropane (MNP).

## Conflict of interest

The authors declare no conflict of interest.

## Supporting information

As a service to our authors and readers, this journal provides supporting information supplied by the authors. Such materials are peer reviewed and may be re‐organized for online delivery, but are not copy‐edited or typeset. Technical support issues arising from supporting information (other than missing files) should be addressed to the authors.

Supplementary

## References

[1a] A. H. Shain, B. C. Bastian, Nat. Rev. Cancer 2016, 16, 345–358; 27125352 10.1038/nrc.2016.37

[1b] C. Garbe, K. Peris, A. Hauschild, P. Saiag, M. Middleton, L. Bastholt, J. Grob, J. Malvehy, Eur. J. Cancer 2016, 63, 201–217. 27367293 10.1016/j.ejca.2016.05.005

[2] C. Garbe, Melanoma Res. 1993, 3, 291–299. 7693093

[3] H. Helmbach, E. Rossmann, M. A. Kern, D. Schadendorf, Int. J. Cancer 2001, 93, 617–622. 11477569 10.1002/ijc.1378

[4] G. Bérubé, Expert Opin. Drug Discovery 2016, 3, 281–305. 10.1517/17460441.2016.1135125 26727036

[5] C. Viegas-Junior, A. Danuello, V. da Silva Bolzani, E. J. Barreiro, C. A. Fraga, Curr. Med. Chem. 2007, 14, 1829–1852. 17627520 10.2174/092986707781058805

[6] L. F. Tietze, H. P. Bell, S. Chandrasekhar, Angew. Chem. Int. Ed. 2003, 42, 3996–4028; 10.1002/anie.200200553 12973759

[7a] F. Gao, Z. Sun, F. Kong, J. Xiao, Eur. J. Med. Chem. 2020, 188, 112044; 31945642 10.1016/j.ejmech.2020.112044

[7b] B. Zhang, Arch. Pharm. 2020, 353, e1900240; 10.1002/ardp.201900240 31797422

[7c] Y. Tu, Nat. Med. 2011, 17, 1217–1220; 21989013 10.1038/nm.2471

[7d] C. Horwedel, S. B. Tsogoeva, S. Wei, T. Efferth, J. Med. Chem. 2010, 53, 4842–4848. 20527917 10.1021/jm100404t

[8a] H. C. Lai, N. P. Singh, T. Sasaki, Invest. New Drugs 2013, 31, 230–246; 22935909 10.1007/s10637-012-9873-z

[8b] T. Fröhlich, A. Ç. Karagöz, C. Reiter, S. B. Tsogoeva, J. Med. Chem. 2016, 59, 7360–7388; 27010926 10.1021/acs.jmedchem.5b01380

[8c] A. Ç. Karagöz, C. Reiter, E. J. Seo, L. Gruber, F. Hahn, M. Leidenberger, V. Klein, F. Hampel, O. Friedrich, M. Marschall, B. Kappes, T. Efferth, S. B. Tsogoeva, Bioorg. Med. Chem. 2018, 26, 3610–3618. 29887512 10.1016/j.bmc.2018.05.041

[9a] C. J. Janse, A. P. Waters, J. Kos, C. B. Lugt, Int. J. Parasitol. 1994, 4, 589–594; 10.1016/0020-7519(94)90150-3 8082988

[9b] A. Çapcı, M. M. Lorion, H. Wang, N. Simon, M. Leidenberger, M. C. Borges Silva, D. R. M. Moreira, Y. Zhu, Y. Meng, J. Y. Chen, Y. M. Lee, O. Friedrich, B. Kappes, J. Wang, L. Ackermann, S. B. Tsogoeva, Angew. Chem. Int. Ed. Engl. 2019, 58, 13066–13079. 10.1002/anie.201907224 PMC6899722 31290221

[10a] T. Efferth, M. R. Romero, D. G. Wolf, T. Stamminger, J. J. Marin, M. Marschall, Clin. Infect. Dis. 2008, 47, 804–811; 18699744 10.1086/591195

[10b] F. E. Held, A. A. Guryev, T. Fröhlich, F. Hampel, A. Kahnt, C. Hutterer, M. Steingruber, H. Bahsi, C. von Bojnicić-Kninski, D. S. Mattes, T. C. Foertsch, A. Nesterov-Mueller, M. Marschall, S. B. Tsogoeva, Nat. Commun. 2017, 8, 15071. 28462939 10.1038/ncomms15071 PMC5418574

[11a] T. Efferth, Curr. Drug Targets 2006, 7, 407–421; 16611029 10.2174/138945006776359412

[11b] T. Fröhlich, C. Mai, R. P. Bogautdinov, S. N. Morozkina, A. G. Shavva, O. Friedrich, D. F. Gilbert, S. B. Tsogoeva, ChemMedChem 2020, 15, 1473–1479. 32374071 10.1002/cmdc.202000174 PMC7496903

[12] L. Botta, S. Filippi, B. M. Bizzarri, C. Zippilli, R. Meschini, R. Pogni, M. C. Baratto, L. Villanova, R. Saladino, ACS Omega 2020, 5, 243–251. 31956771 10.1021/acsomega.9b02600 PMC6964273

[13] A. K. Marković, J. Torić, M. Barbarić, C. J. Brala, Molecules 2019, 24, 2001.

[14] P. M. O'Neill, V. E. Barton, S. A. Ward, Molecules 2010, 15, 1705–1721. 20336009 10.3390/molecules15031705 PMC6257357

[15] L. Botta, S. Filippi, C. Zippilli, S. Cesarini, B. M. Bizzarri, A. Cirigliano, T. Rinaldi, A. Paiardini, D. Fiorucci, R. Saladino, R. Negri, P. Benedetti, ACS Med. Chem. Lett. 2020, 11, 1035–1040. 32435422 10.1021/acsmedchemlett.0c00131 PMC7236541

[16] R. K. Haynes, H. W. Chan, M. K. Cheung, W. L. Lam, M. K. Soo, H. W. Tsang, A. Voerste, I. D. Williams, Eur. J. Org. Chem. 2002, 1, 113–132.

[17] Y. J. Shi, D. L. Hughes, J. M. McNamara, Tetrahedron Lett. 2003, 44, 3609–3611.

[18] S. Kamchonwongpaisan, S. Paitayatat, Y. Thebtaranonth, P. Wilairat, Y. Yuthavong, J. Med. Chem. 1995, 38, 2311–2316. 7608896 10.1021/jm00013a007

[19] T. Fröhlich, C. Reiter, M. E. M. Saeed, C. Hutterer, F. Hahn, M. Leidenberger, O. Friedrich, B. Kappes, M. Marschall, T. Efferth, S. B. Tsogoeva, ACS Med. Chem. Lett. 2018, 9, 534–539. 29937978 10.1021/acsmedchemlett.7b00412 PMC6004568

[20] X. D. Luo, H. J. C. Yeh, A. Brossi, J. L. Flippen-Anderson, R. Gilardi, Helv. Chim. Acta 1984, 67, 1515–1522.

[21a] A. J. Lin, M. Lee, D. L. Klayman, J. Med. Chem. 1989, 32, 1249–1252; 2657065 10.1021/jm00126a017

[21b] P. L. Yu, Y.-X. Chen, L. Ying, R.-Y. Ji, Acta Pharm. Sin. 1985, 20, 357–365;

[21c] A. J. Lin, D. L. Klayman, W. K. Milhous, J. Med. Chem. 1987, 30, 2147–2150. 3669021 10.1021/jm00394a037

[22a] P. Deslongchamps in The Anomeric Effect and Associated Stereoelectronic Effects (Eds: G. R. J. Thatcher), Am. Chem. Soc., Washington D. C., ACS Symp. Ser. 539, **1993**, 26–54;

[22b] N. Pothier, S. Goldstein, P. Deslongchamps, Helv. Chim. Acta 1992, 75, 604–620;

[22c] A. J. Bennett, M. L. Sinnot, J. Am. Chem. Soc. 1986, 108, 7287–7294.

[23] P. M. O′Neill, A. Miller, L. P. D. Bishop, S. Hindley, J. L. Maggs, S. A. Ward, S. M. Roberts, F. Scheinmann, A. V. Stachulski, G. H. Posner, B. K. Park, J. Med. Chem. 2001, 44, 58–68. 11141088 10.1021/jm000987f

[24] B. Neises, W. Steglich, Angew. Chem. Int. Ed. 1978, 17, 522–524;

[25] P. M. O'Neill, A. P. Higson, S. Taylor, E. Irving, WO03/048167.

[26] T. Fröhlich, C. Reiter, M. M. Ibrahim, J. Beutel, C. Hutterer, I. Zeitträger, H. Bahsi, M. Leidenberger, O. Friedrich, B. Kappes, T. Efferth, M. Marschall, S. B. Tsogoeva, ACS Omega 2017, 2, 2422–2431. 30023664 10.1021/acsomega.7b00310 PMC6044832

[27] L. Botta, S. Filippi, B. M. Bizzarri, R. Meschini, M. Caputo, L. Proietti-De-Santis, C. Iside, A. Nebbioso, G. Gualandi, R. Saladino, Bioorg. Med. Chem. Lett. 2019, 29, 78–82. 30442421 10.1016/j.bmcl.2018.11.006

