# Supplementary material for: Stereoselective Access to Antimelanoma Agents by Hybridization and Dimerization of Dihydroartemisinin and Artesunic acid
Source: ChemMedChem. 2021 May 7;16(14):2270–7. doi: 10.1002/cmdc.202100196 (PMC8360007; doi:10.1002/cmdc.202100196)
Supplement: Supplementary file 1 — Supplementary [file CMDC-16-2270-s001.pdf]

ChemMedChem

Supporting Information

Stereoselective Access to Antimelanoma Agents by Hybridization and Dimerization of Dihydroartemisinin and Artesunic acid

Lorenzo Botta,* Silvia Cesarini, Claudio Zippilli, Silvia Filippi, Bruno Mattia Bizzarri, Maria Camilla Baratto, Rebecca Pogni, and Raffaele Saladino*

Table of Contents

S1. Chemistry

S2. Stability experiments

S3. EPR experiments

S1. *Chemistry*

General Part

All reactions were performed in flame-dried glassware under a nitrogen atmosphere. Reagents were obtained from commercial suppliers (Sigma-Aldrich Srl, Milan, Italy) and used without further purification. TLC chromatography was performed on precoated aluminium silica gel SIL G/UV254 plates (Macherey-Nagel & Co.). The detection occurred via fluorescence quenching or development in a molybdato phosphate solution (10% in EtOH). Merck silica gel 60 was used for flash chromatography (23-400 mesh). All products were dried in high-vacuum (10-3 mbar). ^{1}H NMR and ^{13}C NMR spectra were measured on a Bruker Avance DRX400 (400 MHz/100 MHz) spectrometer. Chemical shifts for protons are reported in parts per million (δ scale) and internally referenced to the CD_3OD or $CDCl_3$ signal at δ 3.33 ppm and 7.28 ppm respectively. Mass spectra (MS) data were obtained using an Agilent 1100 LC/MSD VL system (G1946C) with a 0.4 mL/min flow rate using a binary solvent system of 95:5 methyl alcohol/water. UV detection was monitored at 254 nm. Mass spectra were acquired in positive and negative mode scanning over the mass range. Elemental analyses (C, H, N) were performed in house. Optical rotations were recorded on a JASCO P-1000 series at 589 nm and reported as follows: $[\alpha]_D$ = value (concentration in g/100 mL, solvent). Artemisinin, dihydroartemisinin and artesunate were obtained from Lachifarma s.r.l. (Zollino (LE), Italy.

Procedure for the synthesis of derivative ***16α****:*

To a solution of dihydroartemisinin **2** (300 mg, 1.05 mmol, 1 equiv.) in dry DCM (15 mL), phtalic anhydride (187 mg, 1.26 mmol, 1.2 equiv.) and Et_3N (525 µL, 5.25 mmol, 1 equiv.) were added. The solution was stirred for 1 hour at room temperature. After this period, the reaction mixture was washed with aqueous solution of citric acid (0.1 M, pH 2, 20 mL) and brine (20 mL), dried over Na_2SO_4, filtered and concentrated under reduced pressure. The crude product was purified by flash column chromatography (Hex/ EtOAc 1:1).

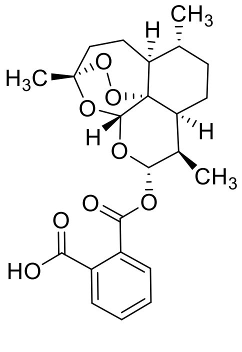

Yield = 66%. R_f = 0.20 (Hex/EtOAc 1:1, molybdato phosphate). ^{1}H-NMR ($CDCl_3$, 400 MHz): δ = 7.90-7.87 (m, 2H), 7.62-7.59 (m, 2H), 6.02 (d, 1H, J = 10.0 Hz), 5.54 (s, 1H), 2.69-2.67 (m, 1H), 2.40-2.37 (m, 1H), 2.08-2.04 (m, 1H), 1.95-1.87 (m, 1H), 1.86-1.83 (m, 1H), 1.78-1.74 (m, 1H), 1.69-1.66 (m, 1H), 1.51-1.47 (m, 1H), 1.46 (s, 3H), 1.39-1.26 (m, 3H), 1.09-1.06 (m, 1H), 1.00-0.99 (m, 3H), 0.95-0.93 (m, 3H) ppm. ^{13}C-NMR ($CDCl_3$, 100 MHz): δ = 168.5, 166.3, 132.2, 131.8, 129.3, 129.0, 104.1, 93.4, 91.2, 80.4, 79.6, 56.5, 51.6, 49.0, 45.2, 36.7, 36.5, 34.2, 26.0, 24.8, 21.5, 20.5,

12.4 ppm. MS (ESI): *m/z* for $[C_{23}H_{27}O_8]^-$= 431. Anal. calcd. for $C_{23}H_{28}O_8$: C, 63.88; H, 6.53 O, 29.60; found: C, 63.86; H, 6.54; O, 29.62

S2. Stability experiments

Material and Methods

Compound **22α,α** (10 mg) was dissolved in the cell culture medium (10 mL) under gentle magnetic stirring, and the mixture was left for 24 h and 48 h at room temperature. At the end the cell culture medium (0.5 mL) was extracted with ethyl acetate (0.5 mL) and the organic phase was analyzed by UHPLC Thermo Scientific Dionex Ultimate 3000 equipped with multi-wave length detector. The column used was a Thermo Scientific Hypersil GOLD (15.0 cm length, 4.6 mm I.D., 3 µm particle size) eluted with mixtures of water (solvent A) and acetonitrile (solvent B) at a flow rate of 0.5 mL/min. Elution was performed with isocratic method 5% A and 95% B. Detection was at 220 nm and runtime was set at 10 min. The samples were compared with pure standards of the two compounds. Co-injection experiments were also conducted in order to evaluate the difference in the retention time of the two derivatives artesunic acid **3** (artesunate in the chromatograms' legend) and **22α,α**. As reported in Figure S#1-5 very little degradation of **22α,α** occurred in the reported experimental conditions.

S#1. **HPLC analysis of pure artesunic acid 3.**

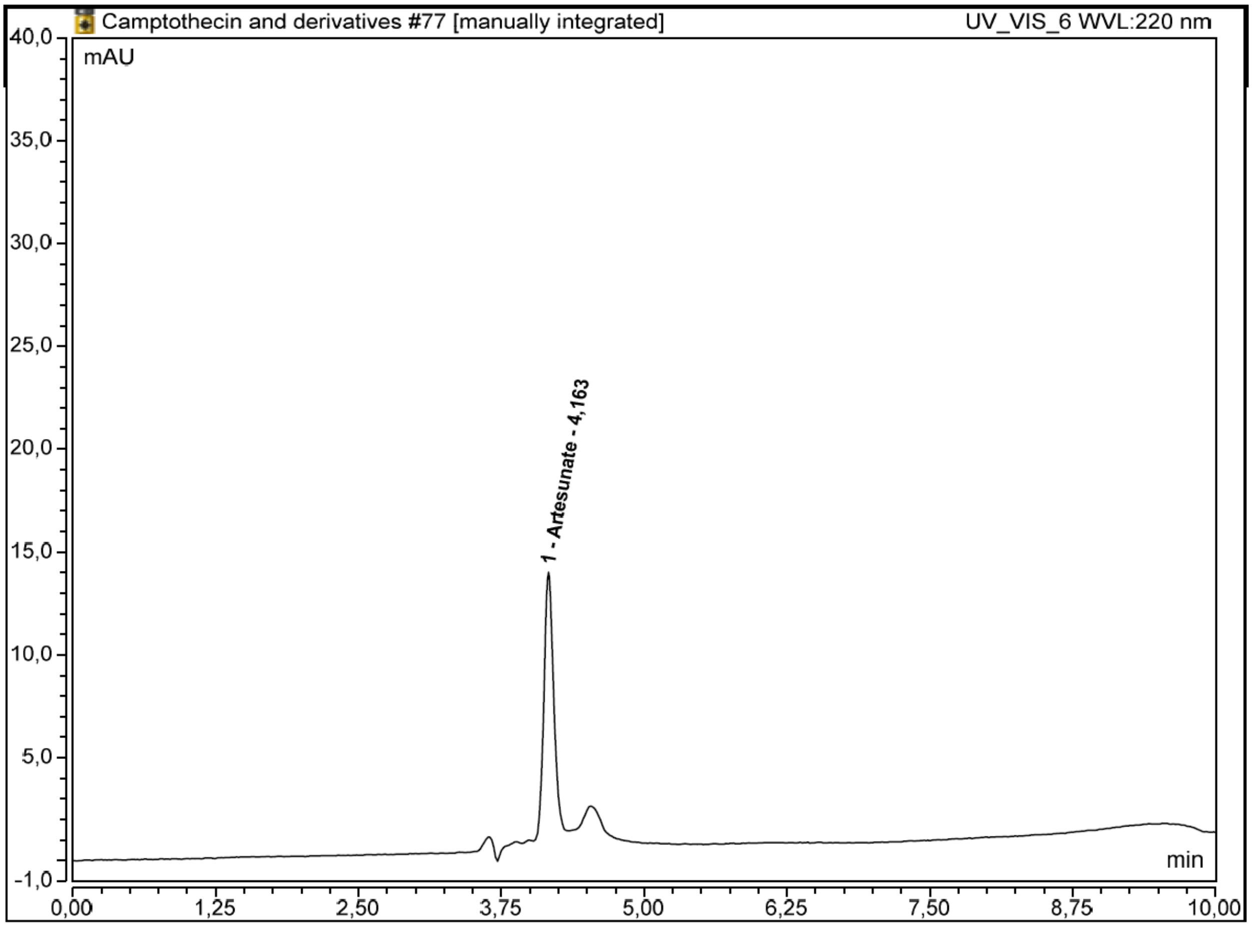

S#2. HPLC analysis of pure 22α,α.

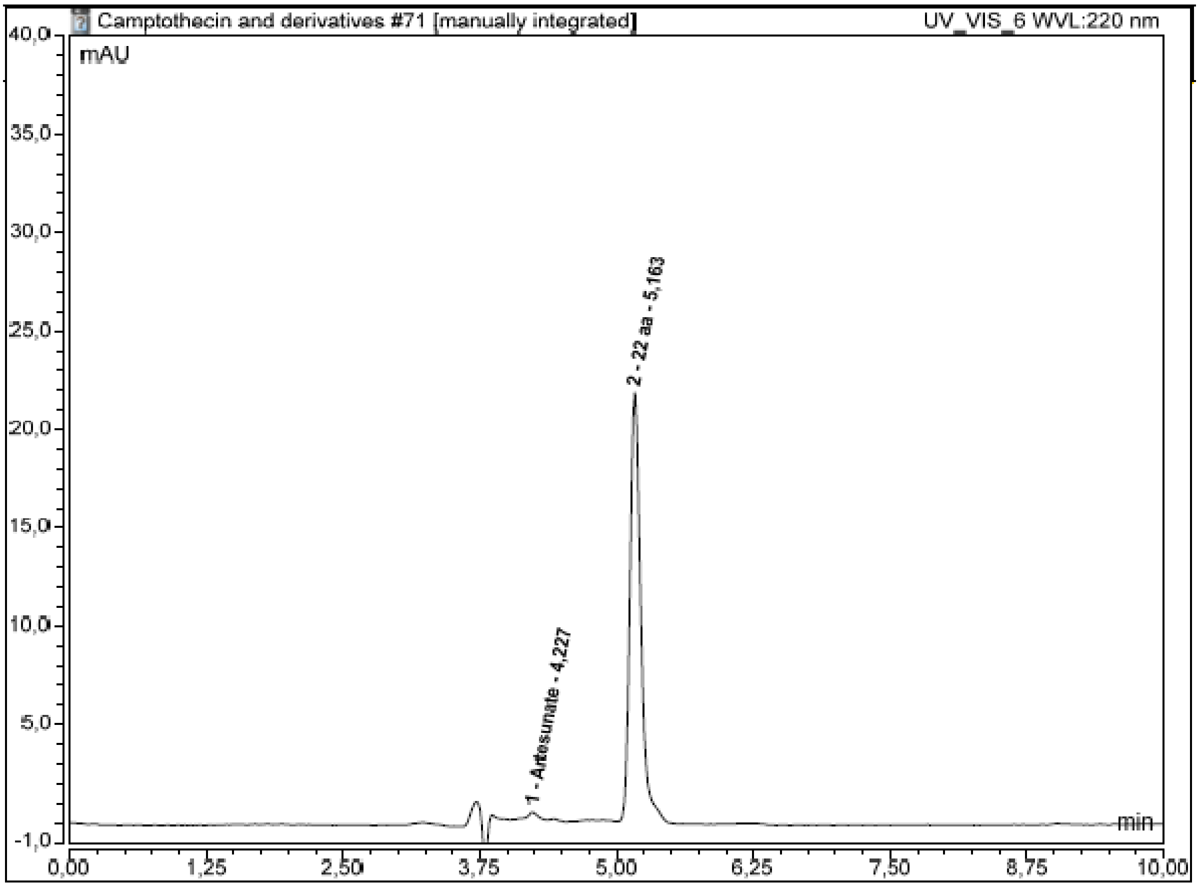

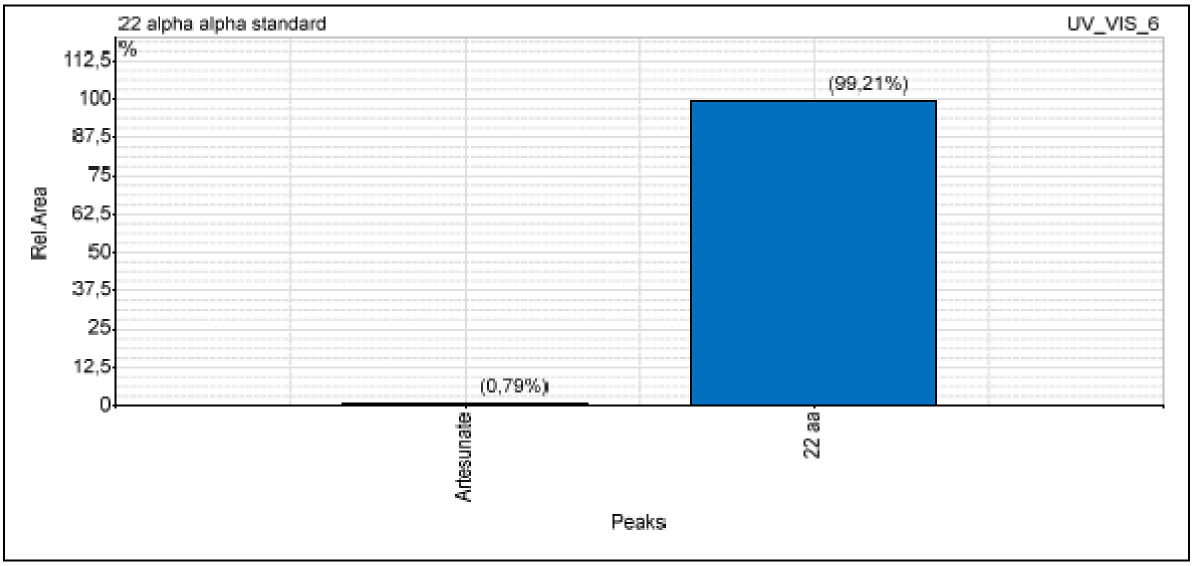

S#3. HPLC co-injection analysis of artesunic acid 3 and derivative 22α,α.

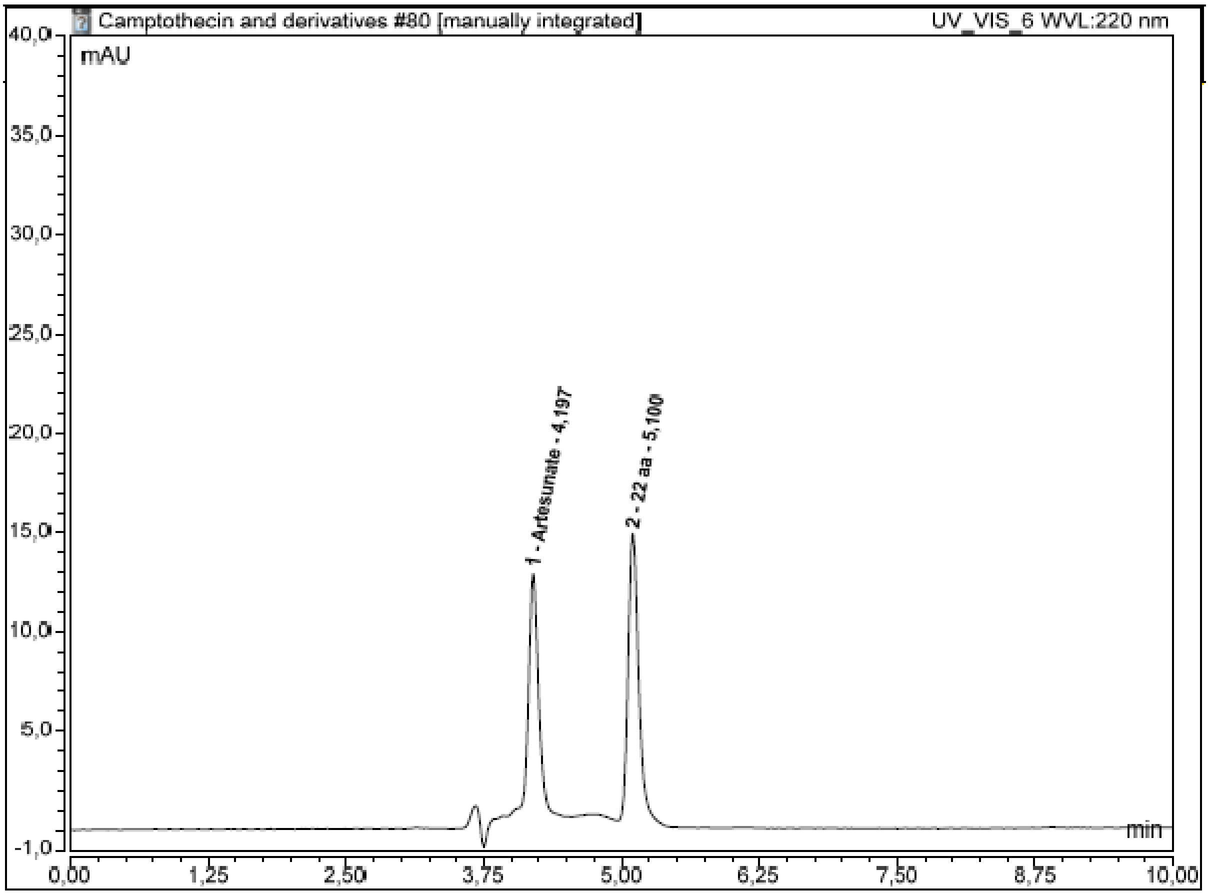

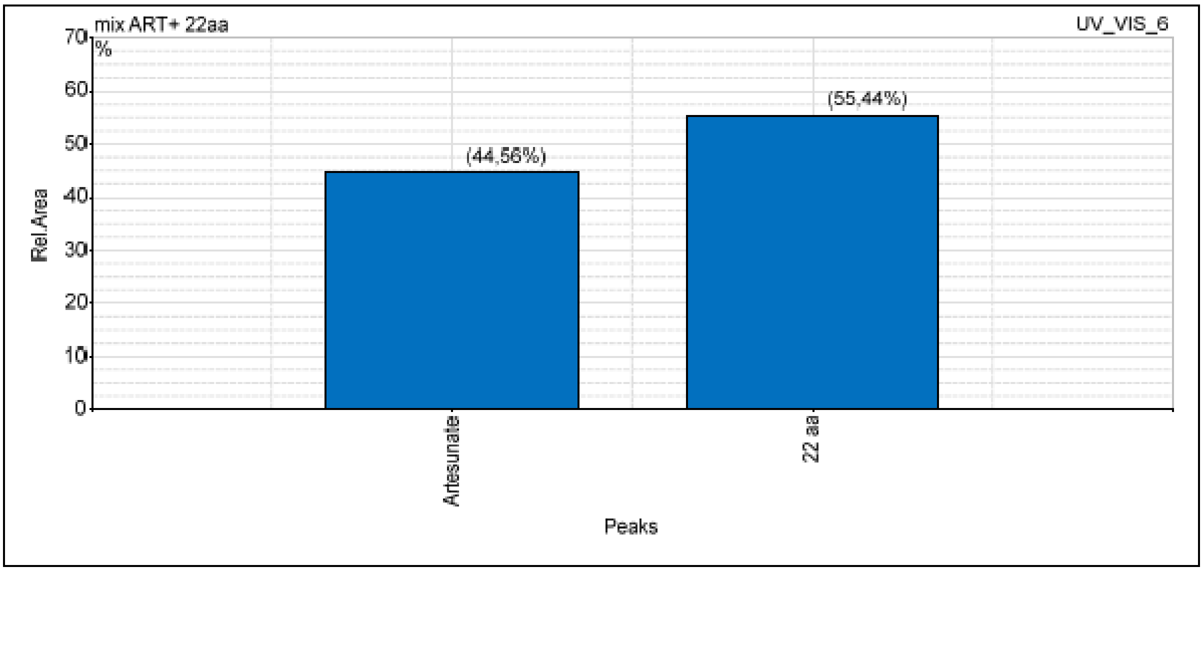

S#4. HPLC analysis of 22α,α after 24 h.

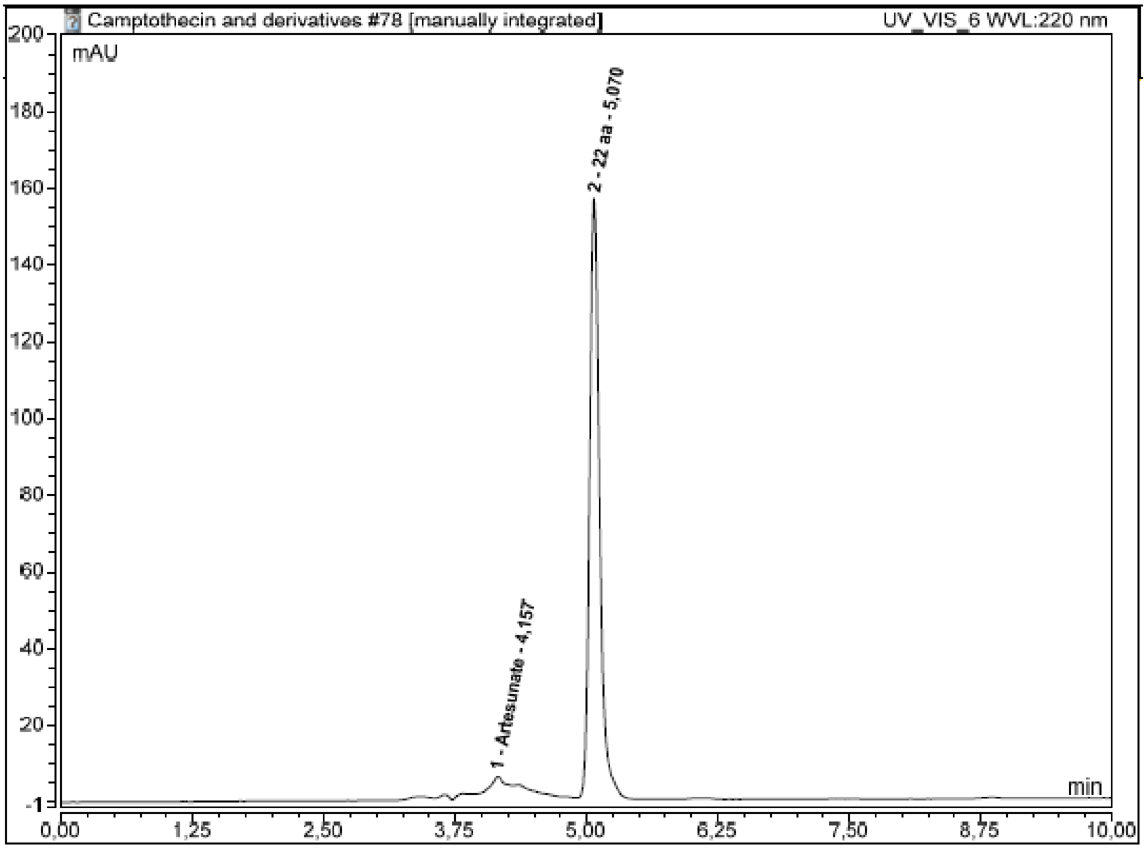

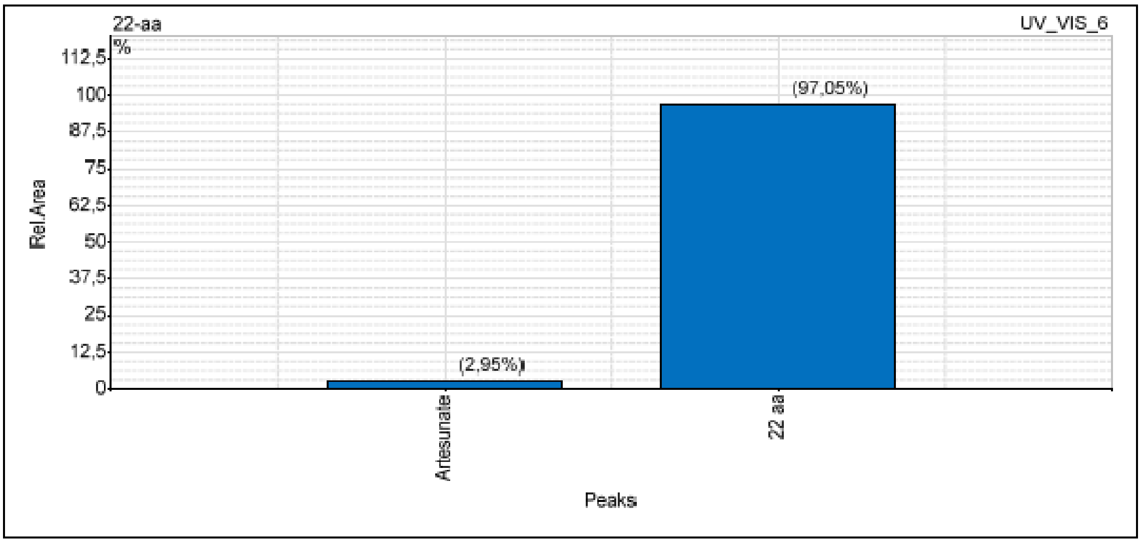

S#5. **HPLC analysis of 22α,α after 48h.**

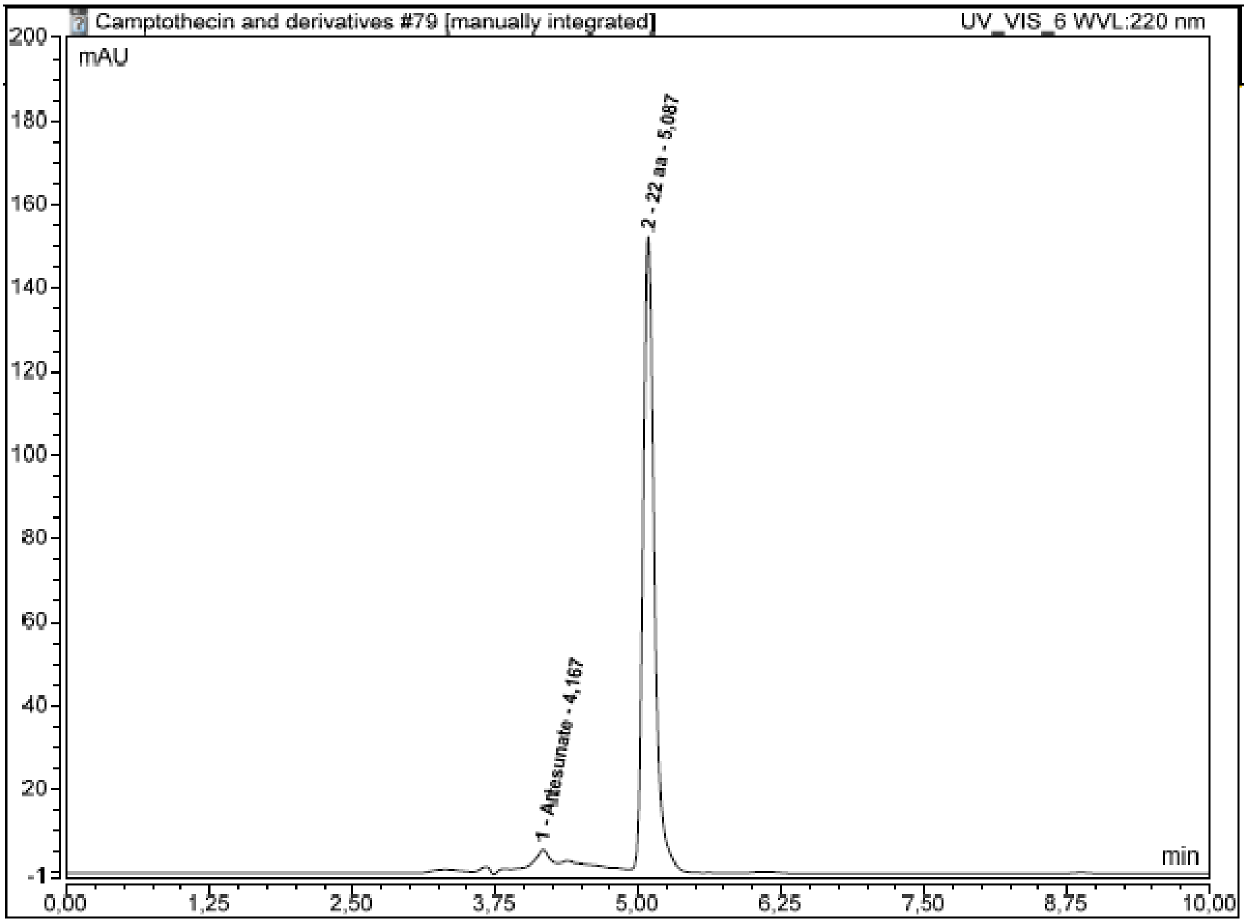

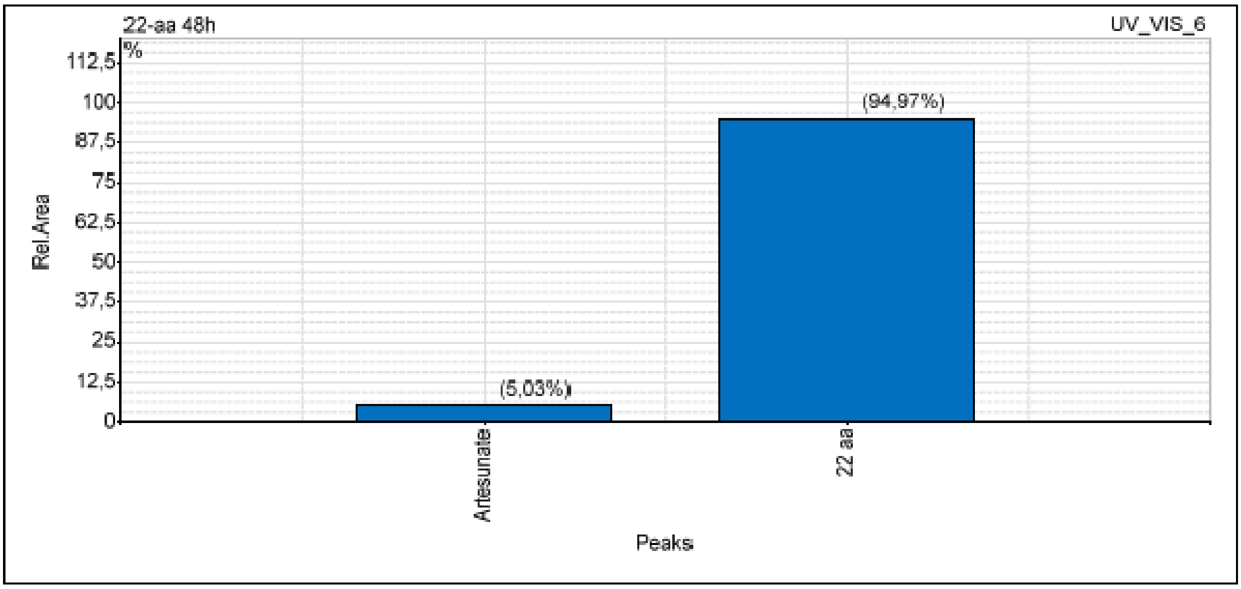

S3. *EPR experiments*

Material and Methods

Continuous wave (CW) X-band (9GHz) EPR measurements were carried out with an E580 Elexsys Series spectrometer (Bruker Biospin GmbH, Rheinstetten, Germany) equipped with a Bruker ER 049X microwave bridge and a high sensitivity 4122SHQE/0208 cavity. To perform EPR experiments capillaries of 1.2 mm diameter were filled in and inserted in a quartz tube of 3x3.5 I.D.xO.D. The reaction solution was prepared adding **20αα**, **21αα**, **22αα** 12mM, MNP 42mM and Fe(II)SO_4 5mM all dissolved in a acetonitrate:water solution 50%. Fe(II)SO_4 is the last addition as it is the catalyst that starts the reaction. Experimental conditions are: 0.1 mT modulation amplitude, 0.3 mW microwave power, 9.864 GHz microwave frequency. The spectra were recorded at t = 0 (considered after the addition of Fe(II)SO_4 to the solution) reaction time and at different times after the reaction was started.
In the following figures are reported EPR spectra of the **20-22-αα** in the presence of Fe(II)SO_4 and the spin trap MNP recorded at different times [t = 0 min, t = 15 min, t = 70 min, t = 120 min, t = 150 min and t = 180 min in respect to the addition of the last reagent Fe(II)SO_4]. All the spectra are compared with that obtained adding the MNP to the products **20-22-αα** in order to verify if the radical production is induced by the Fe(II)SO_4 addition and it is not formed before.

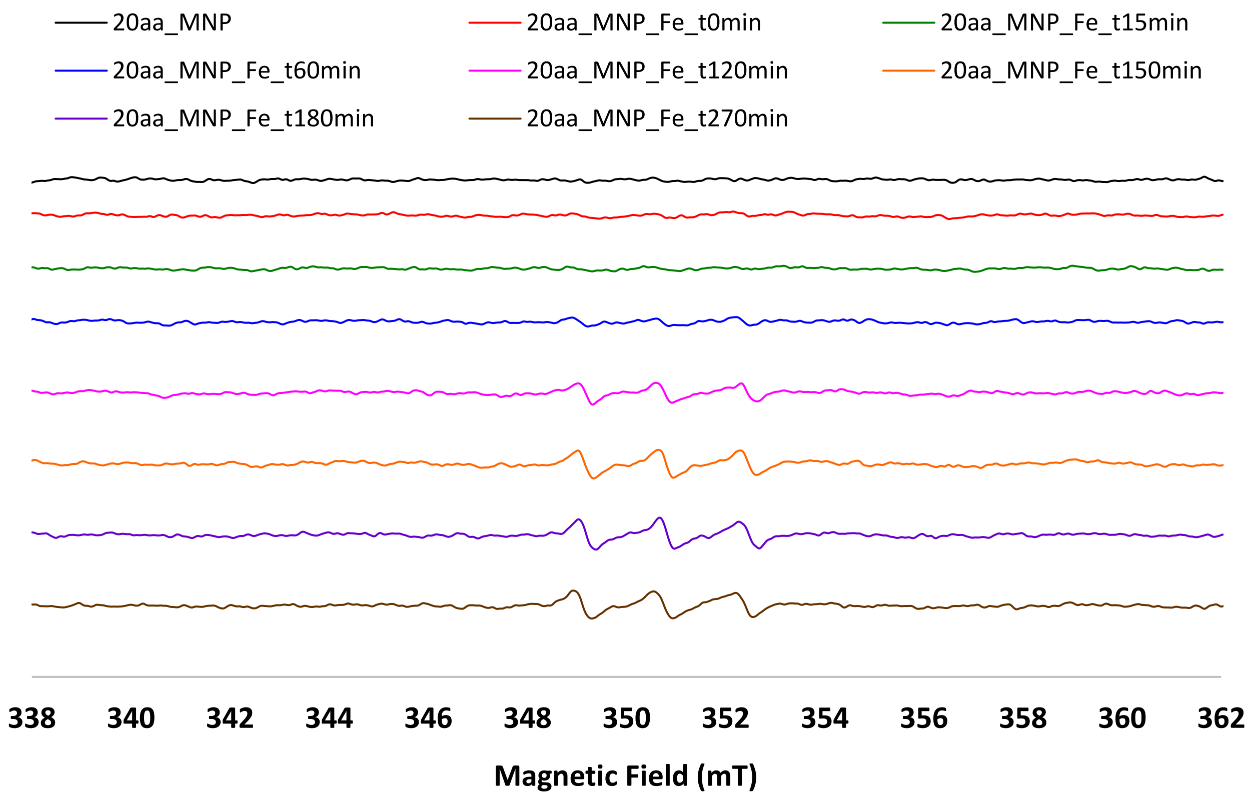

Figure S#6. X-band EPR spectra of the radical species formed during the reaction of **20-αα**, MNP and Fe(II)SO_4 at different times from 0 minute to 270 minutes after the addition of the last reagent Fe(II)SO_4. *Experimental condition.* 9.866 GHz microwave frequency, 0.1 mT modulation amplitude and 0.2 mW microwave power.

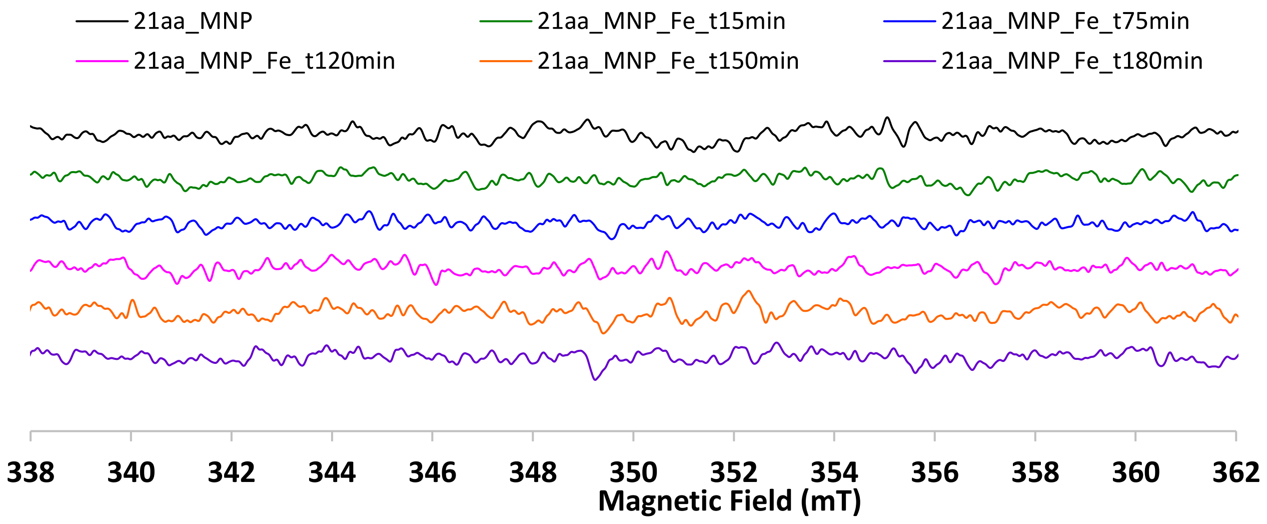

Figure S#7. X-band EPR spectra of the reaction with **21-αα**, MNP and Fe(II)SO_4 at different times from 15 minute to 180 minutes after the addition of the last reagent Fe(II)SO_4. *Experimental condition.* 9.866 GHz microwave frequency, 0.1 mT modulation amplitude and 0.2 mW microwave power.

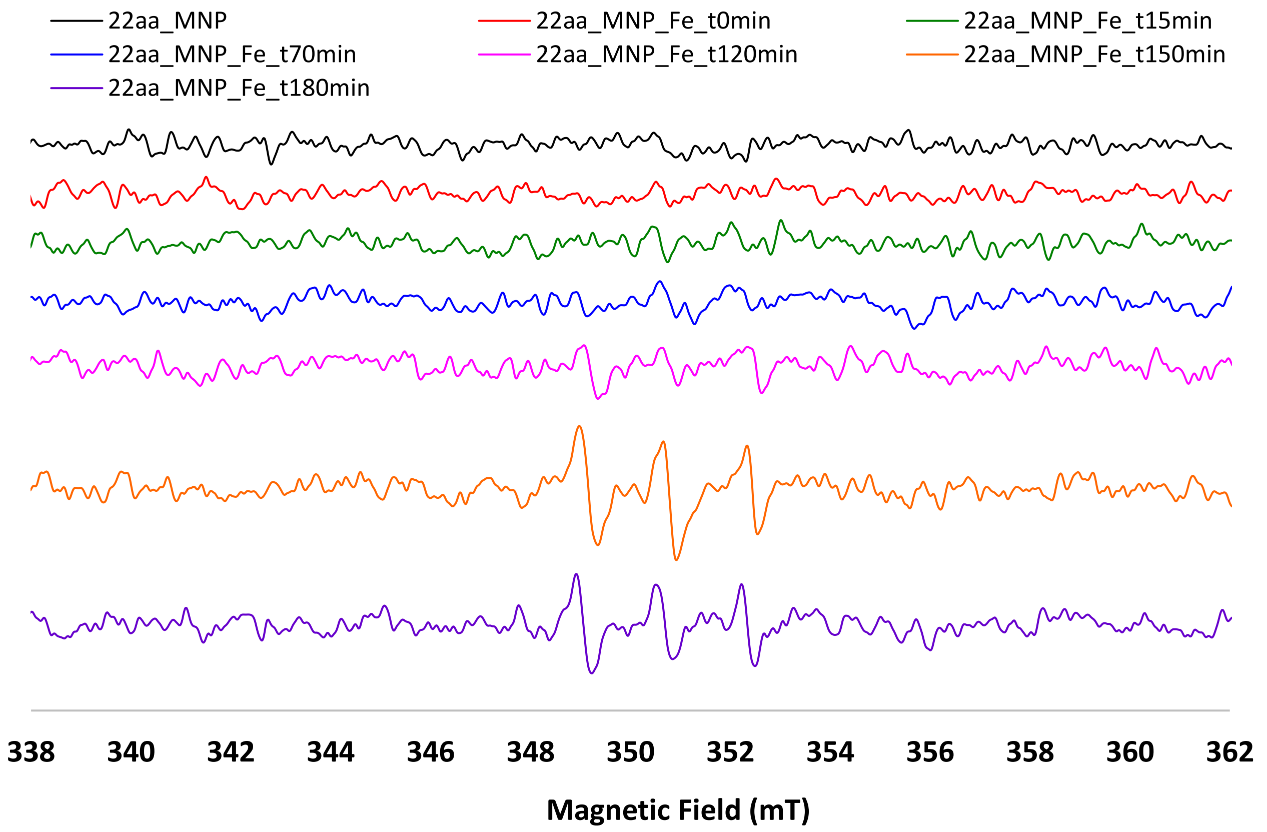

Figure S#8. X-band EPR spectra of the reaction with **22-αα**, MNP and Fe(II)SO_4 at different times from t = 0 minute to 180 minutes after the addition of the last reagent Fe(II)SO_4. *Experimental condition.* 9.866 GHz microwave frequency, 0.1 mT modulation amplitude and 0.2 mW microwave power.

Abbreviation

3-(4,5-dimethylthiazol-2-yl)-2,5-diphenyltetrazolium bromide (MTT); triphenylphoshine (PPh_3), diisopropyl azodicarboxylate (DIAD), *N*,*N*-dicyclohexylcarbodiimide (DCC); dimethylaminopyridine (DMAP); 1-Hydroxybenzotriazolehydrate (HOBt); *N*,*N*-diisopropylethylamine (DIPEA); *N*-ethyl-*N*'-3-(dimethylaminopropyl)carbodiimidehydrocloride (EDC · HCl); boron trifluoride dietherate ($BF_3 \cdot OEt_2$); triethylamine (Et_3N); potassium carbonate (K_2CO_3); ethylacetate (EtOAc); hexane (Hex); dichloromethane (DCM); *N*,*N*-dimethylformamide (DMF); methanol (MeOH); deferoxamine (DFO); Electron paramagnetic resonance (EPR); 2-methyl-2 nitrosopropane (MNP).
